# Spin Coherence and Electron Spin Distribution of a Silver(II) *S* = 1/2 Molecular System

**DOI:** 10.1021/acs.inorgchem.5c00203

**Published:** 2025-06-11

**Authors:** Joan Serra, Enrico Salvadori, Yu-Kai Liao, Albert Gallén, Albert Escuer, Mario Chiesa, Júlia Mayans

**Affiliations:** † Departament de Química Inorgànica i Orgànica Secció de Química Inorgànica, 430160 Universitat de Barcelona, Marti i Franques 1-11, Barcelona 08028, Spain; ‡ Department of Chemistry and NIS Centre, 9314 University of Torino, Via Giuria 7, Torino 10125, Italy; § Institut de Nanociència i Nanotecnologia (IN2UB), Universitat de Barcelona, Barcelona 08028, Spain

## Abstract

The spin–lattice relaxation time, spin coherence, and spin distribution have been studied through ac susceptometry, pulse EPR, and ultralow-frequency Raman spectroscopy on a silver­(II)-derived molecular system with spin 1/2. The combination of magnetometry and spectroscopy techniques demonstrates the occurrence of slow spin magnetic relaxation induced by a spin–phonon interaction. The magnetic behavior and the spin coherence of this Ag^II^-derived system open the door to a new cation into the scarce family of *S* = 1/2 slow magnetic relaxing systems for further applications in quantum technologies.

## Graphical abstract

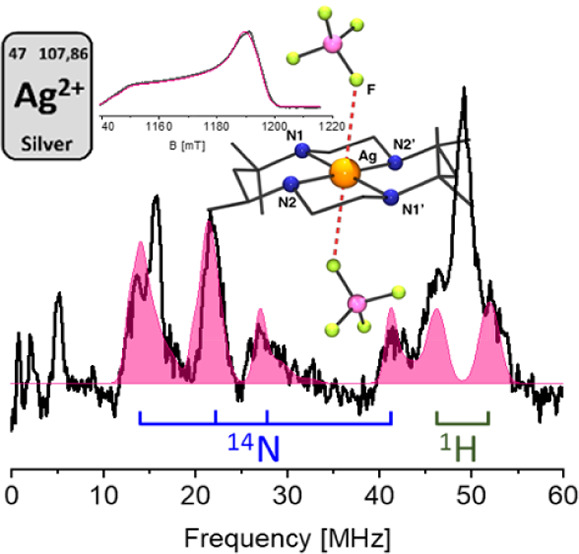

## Introduction

One of the main goals in exploring the nanoworld is the development of devices working under the laws of quantum mechanics. Quantum superposition, quantum entanglement, and quantum coherence, highly connected capital concepts, have been exploited in recent years in the so-called quantum coherent nanoscience (QCN)^1^ and used mainly to develop devices for quantum computing and quantum sensing.^2,3^ Both applications must fully control the creation, behavior, and interaction of their basic building blocks, known as quantum bits (qubits), named for their equivalence with their classical bit counterparts. Coherence times (denoted as *T*_2_ or *T*_m_, the spin–spin relaxation, and the phase memory time, respectively) determine the stability and reliability of quantum information processing and represent the duration for which qubits retain their superposition and entanglement states. Fortunately, any two-level system that fulfills the seven DiVincenzo criteria,^4^ including some paramagnetic molecules, can behave as a qubit and can be initialized through different external stimuli. For this reason, since some years ago, magnetic molecules have been proposed to be used in quantum information processing (QIP).^5–10^ It is known that the use of molecules over other proposed physical systems like electronic defects in silicon carbide^11^ or diamond,^12^ allows chemical tunability of their electronic properties, involving, obviously, a tunability in the magnetic features. For this purpose, electronic spins in molecular systems can be used if control of the transitions between states is available. In the case of electronic spin, *S* = 1/2 could be quickly addressed by electromagnetic fields, and, in some cases, they present coherence times long enough to allow single-qubit gate manipulation.^13^ The easy addressability of electron spins implies that they are also very sensitive to their environment (in chemical terms, it means other spins, ligands, or solvent molecules), and different strategies have been followed to enhance their coherence times, including the removal of nuclear spins in the vicinity of the potential qubits^14^ and, recently, some publications have explored the combination of alternating current (ac) susceptometry measurements with different electron paramagnetic resonance (EPR) techniques to identify molecules with slow spin–lattice relaxation, focusing the efforts to increase the spin–lattice relaxation time (*T*_1_), which is the upper limit of *T*_2._ ^6,7^

Slow relaxation of the magnetization and/or single-qubit application of *S* = 1/2 systems have been recently studied in several cations,^15–29^ such as Cu^II^, Ni^III^, Mn^IV^, or more extensively VO^II^, for which it has been suggested that the relaxation mechanisms involve low-energy vibrational modes that can be modulated by employing ligands with different degrees of rigidity.^30,31^

Silver chemistry is dominated by its monovalent cation that is diamagnetic as a consequence of its 4*d* ^10^ electronic configuration. The coordination chemistry of the elusive paramagnetic Ag^II^ cation, having 4*d* ^9^ configuration, is very limited due to its very strong oxidizing character that, mainly in solution, prevents stable complexes with most ligands,^32,33^ and its magnetic properties are poorly explored. Aromatic N-donors, such as pyridine or substituted R-py ligands, yield a series of poorly stable complexes with [AgL_4_]^2+^ formula,^34–36^ whereas systems with [AgL_2_]^2+^ formula for L = bipyridine or phenanthroline exhibit better stability.^37–39^ Aliphatic tetraaza macrocyclic ligands, such as cyclam or 5,5,7,12,12,14-hexamethyl-cyclam (CTH) (Scheme 1), provide stable complexes that can be obtained from the spontaneous dismutation of Ag^I^ in the presence of these macrocycles, yielding [AgL]­X_2_ complexes, in which the cation exhibits the expected elongated octahedral arrangement due to the strong Jahn–Teller effect, linking the X^–^ counteranions on the elongated axial sites.^40–42^

**Scheme 1:**
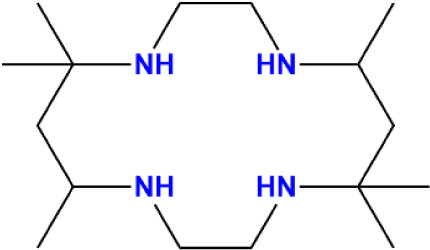
5,5,7,12,12,14-Hexamethyl-Cyclam (CTH) Ligand, Used in This Work

Dynamic properties for the Ag^II^ cation were partially explored for one Ag^II^-tetratolylporphyrin complex by pulsed EPR in 1996 ^43^ and the ac susceptibility response has been pointed out in a recent paper of the authors for the complexes [Ag­(*meso*-CTH)]­X_2_, where X = NO_3_^–^ or ClO_4_^–^ counteranions, suggesting the occurrence of slow spin relaxation of the magnetization induced by spin–phonon interactions.^44^ The complex with the less coordinating perchlorate anion gave clearly better stability and clearer out-of-phase signals than its nitrato analogue, suggesting that the reduction of the axial interaction improves the properties of the system.

On this basis, employing the *meso*-CTH macrocyclic ligand, which gives a good isolation between the paramagnetic centers due to the presence of the six peripheral methyl groups and the poorly coordinating tetrafluoroborate anion, we have prepared the new [Ag­(*meso*-CTH)]­(BF_4_)_2_ (**1**) complex that exhibits excellent SRM response. The system has been characterized by single-crystal X-ray determination in the solid state and NMR in solution. Complete spin dynamics were studied by means of ac magnetometry and pulsed EPR.

## Experimental Section

### X-ray Crystallography

Orange prism-like specimens of **1** were used for the X-ray crystallographic analysis. The X-ray intensity data were measured on a D8 Venture system equipped with a multilayer monochromator and a Mo microfocus.

The frames were integrated with the Bruker SAINT software package using a narrow-frame algorithm. The structures were solved and refined using the Bruker SHELXTL software.^45^

Crystal data and refinement details for complex **1** are summarized in Table S1. Further crystallographic details can be found in the corresponding CIF file provided in the Supporting Information.

### Physical Measurements

Magnetic susceptibility measurements were carried out on pressed polycrystalline samples with an MPMS5 quantum design susceptometer working in the range of 30–300 K under magnetic fields of 0.3 T and under a field of 0.03 T in the 30–2 K range to avoid saturation effects at low temperature. Diamagnetic corrections were estimated from Pascal Tables.^46^ Infrared spectra (4000–400 cm^–1^) were recorded from KBr pellets on a Bruker IFS-125 FT-IR spectrophotometer. UV–vis spectra were recorded with a Varian Cary-100 spectrophotometer. Ultralow frequency Raman spectra were recorded with a high-resolution Raman T64000 (Jobin Yvon) instrument.

Q-band pulsed-EPR experiments were performed on a Bruker Elexsys E580 spectrometer equipped with a dielectric ring resonator (EN 5107D2) housed in a Cryogenic cryogen-free variable temperature cryostat and a Bruker AmpQ 10 W solid-state amplifier. Solutions of **1** (0.5 mM) in CH_2_Cl_2_/toluene 3:1 v/v were prepared. During the measurements, the resonator was overcoupled to minimize ringdown following the application of the microwave pulses. ESE-detected EPR spectra were measured at *T* = 10 K using a Hahn echo sequence ( π/2−τ−π−τ−echo , with τ = 200 ns and π/2 = 16 ns) at different magnetic fields. EDNMR experiments^47^a,b were performed using a Bruker SpinJet-AWG. Spectra were obtained with the pulse sequence: HTA – *T* – π/2 – τ – π – τ – echo with *t*_HTA_ = 9000 ns, *T* = 1000 ns, *t*_π/2_ = 400 ns, and τ = 600 ns. A Gaussian-shaped ELDOR pulse was used. The integration width of the echo was set to 800 ns, centered around the maximum of the spin echo. The central hole at Δν = 0 was removed in the EDNMR spectra by subtracting a fitted Lorentzian line shape. Simulation of the EDNMR spectra was performed using a simulation algorithm based on the work by Cox et al.^48^ within the EasySpin toolbox.^49^

Coherence times were measured using the Hahn echo sequence with incremented τ . Spin–lattice relaxation times were measured using inversion recovery sequence $\pi -t_{w}-\pi /2-\tau -\pi -\tau -\mathrm{echo}$ with incremented waiting time $t_{w}$ and τ = 200 ns pulse length. The uncertainty in *T*_1_ estimated from replicate measurements was 5–10% depending upon the signal-to-noise ratio at a given temperature-field combination.

^1^H NMR (400 MHz) and ^19^F-NMR (376.5 MHz) were acquired on a Bruker 400 MHz spectrometer equipped with a nitrogen cryoprobe at 298 K. ^1^H NMR experiment of the paramagnetic complex **1** was performed using the pulse program zg30, with P1 = 11.13 μs @ PLW1 = 9.8 W, D1 = 1 s, and AQ = 0.25 s, in order to account for the fast relaxation triggered by the paramagnetic metallic center.

### Synthesis

The *meso*-CTH ligand was prepared according to previously reported methods.^50^

Complex **1** is [Ag^II^(*m*-CTH)]­(BF_4_)_2_. *m*-CTH (0.2 g, 0.6 mmol) and AgBF_4_ (0.24 g, 1.2 mmol) were dissolved in 10 mL of MeCN under stirring during 1 h. The formation of metallic silver was observed immediately. After that time, the mixture was centrifuged before decanting the yellow solution. The metallic silver remaining on the bottom of the tube was washed with 5 mL of MeCN, centrifuged, and the filtrate was added to the first fraction decanted before. Single crystals of **1** suitable for single-crystal X-ray diffraction analyses were obtained by diffusion of diethyl ether into a vial containing an acetonitrile solution of **1**. CHN calc/found: C, 33.95/34.0; H, 6.41/6.3; N, 9.90/9.9.

The IR spectrum is shown in Figure S1. Electronic spectra: the solid-state electronic spectrum of complex **1** shows an absorption centered at 394 nm. This absorption is shown to be sensitive to the axially coordinated counteranions, as can be seen if the spectrum is compared in the solid state with the previously reported perchlorate complex, which shows the absorption displaced to 381 nm (Figure S2). In contrast, the spectra become identical in methanolic solution evidencing the loss of the weakly interacting axial ligands, and exhibiting an absorption at 346 nm with a weaker shoulder around 275 nm in good agreement with previously reported spectra for Ag^II^ macrocyclic derivatives.^51^

^1^H NMR (400 MHz, CDCl_3_, 298 K) for the *m*-CTH ligand and complex **1** and ^19^F-NMR (376.5 MHz, CDCl_3_, 298 K) are reported in Figures S3–S7. No reliable signals were observed in ^11^B-NMR.

## Results and Discussion

### Structural Description

The structure consists of a centrosymmetric mononuclear system in which the Ag^II^ cation is coordinated by one *m*-CTH ligand and two BF_4_^–^ counteranions. A view of the molecular structure is shown in Figure 1 and selected bond parameters are summarized in Table S2. As a consequence of the *d* ^9^ configuration of the Ag^II^ cation and the concomitant Jahn–Teller effect, the Ag^II^ cation is placed in an elongated octahedral coordination environment exhibiting a *trans* arrangement.

**Figure 1:**
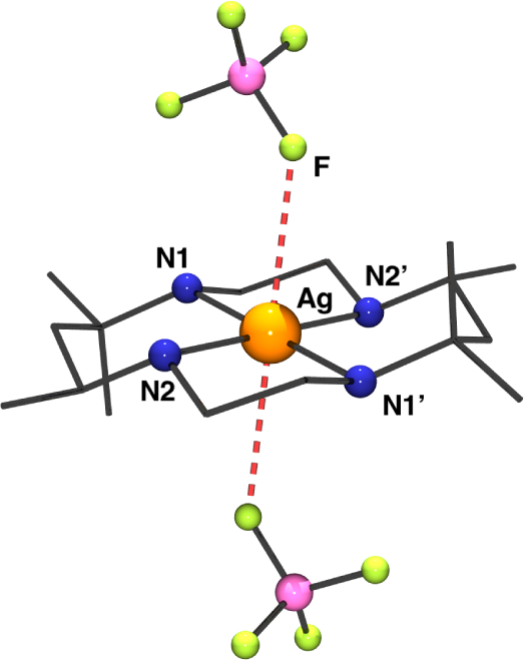
Labeled plot of the Ag^II^ environment for complex **1**.

The four N-donor atoms from the *m*-CTH ligand determine the equatorial plane of the elongated octahedron with the Ag^II^ cation strictly placed on this plane. The four Ag–N bond distances are very close, comprised between 2.156(2) and 2.160(1) Å. The axial coordination sites are occupied by two F-donors coming from the BF_4_^–^ anions, which complete the coordination sphere, weakly interacting with the cation (Ag–F bond distance 2.879(1) Å). The paramagnetic centers are well isolated by the macrocyclic ligand, resulting in a shorter Ag^II^···Ag^II^ intermolecular distance of 8.214(1) Å. The network packing of **1** is depicted in Figure S8 and the coordination sphere and SHAPE^52^ analysis of the Ag^II^ environment are shown in Figure S9.

### Magnetic Measurements

Direct current magnetic susceptibility (χ_M_) data for **1** were measured on polycrystalline and pressed samples in the 2–300 K temperature range and plotted as χ_M_ *T* vs *T* (Figure S10). χ_M_ *T* value at room temperature for complex **1** is 0.395 cm^3^ K mol^–1^, very close to the corresponding spin-only value for the isolated *S* = 1/2 Ag^II^ cation of 0.375 cm^3^ K mol^–1^. The magnetization follows a Brillouin function, tending to 1 Nμ_B_, and χ_M_ *T* follows the expected Curie law along the whole range of temperature (Figure S10, inset).

Initial alternating current measurements showed that no signals were found for **1** at zero field, as expected for an *S* = 1/2 system, but measurements under static increasing magnetic fields revealed well-defined χ_M_″(*T*) peaks in a wide range above 5 K, which revealed to be strongly field dependent (Figure S11).

Measurements under a weak field (0.05 T) revealed a well-defined set of frequency-dependent signals (Figure 2). In light of these previous measurements, the out-of-phase response of **1** was extensively studied in the temperature range of 2.2–50 K under different strengths of the applied magnetic field (0.3, 0.1, 0.3, 0.6, 1.0, 1.5, 2.0, 2.5, and 3.0 T) to relate the different relaxation paths with temperature and the applied field (Figure S12).

**Figure 2:**
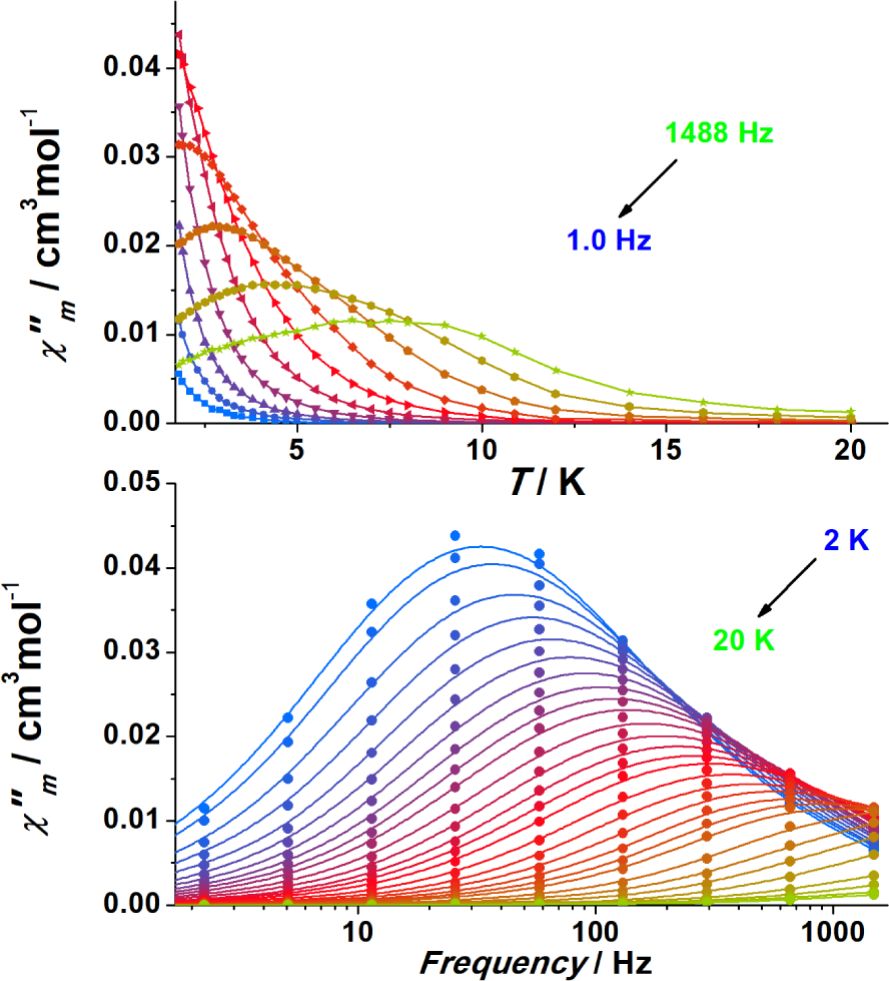
Temperature and frequency dependence of the out-of-phase response of complex **1** under an external applied field of 0.05 T. Complete measurements between 0.3 and 3 T are shown in Figure S12.

The experimental data *χ′* and *χ″* at different magnetic fields of **1** were represented in the Argand plots (Figure S13) and fitted using a self-made program with the generalized Debye model^53^ to extract the relaxation time dependence with temperature (Figure 3 and Table S3). A new maximum in *χ″*(*ν*) (Figure S12) and a semicircle in the Argand plots (Figure S13) are detected in the low-temperature region (2.3–6 K) above 1 T, indicating the contribution of a new relaxation pathway that disappears in the high-temperature region (6.5–40 K). Two independent fittings have been performed in each temperature region. For simplicity, only the τ values obtained from the fittings in the high-temperature region above 1 T have been considered in further analyses. It is clear from the logarithmic representation of τ vs *T* (Figure S14) that there is a very small change in the slope of the curves (values are reported in Table S4). Indeed, it indicates a change in the nature of the main relaxation path in the higher fields region (1.5–3 T), and because of the intrinsic nature of **1** (*S* = 1/2), the relaxation could not follow an Arrhenius law for the magnetic relaxation, and the data have been fitted using eq 1:

1 $$\tau^{-1}=aT+bT^{n}$$

with two contributions to the relaxation: the first term corresponds to the direct mechanism, which dominates at low temperature, and the second term is the Raman multiphonon process through virtual states, a thermally activated process.^54^ This model reproduces the experimental behavior, with different contributions of each one depending on the strength of the applied magnetic field: at low fields, only the Raman process is active, while with increasing field, the direct relaxation process appears (Figure S15). Best-fitting parameters are reported in Table 1. Interestingly, the value of the Raman exponents is *n* < 3, much smaller than the predicted values for Kramers ions and deviated from the expected in a phonon-bottleneck occurrence. However, similar values of the Raman exponents have been reported for other *S* = 1/2 systems recently^7,8^ indicating the intervention of acoustic (lattice) and optical (molecular) phonons, which is possible because there is no significant axial anisotropy in **1**.^55^

**Table 1:** Best Fit Parameters for 1 Used to Reproduce the Relaxation Rate Dependence with the Temperature

*B*(T)	*C*(s^–1^K^–n^)	*n*	*A*(s^–1^K^–1^)	*T*range (K)
0.05	119(2)	2.09(1)	-	2.3–12
0.1	57.3(3)	2.198(3)	-	2.3–18
0.3	12.4(3)	2.31(1)	-	2.3–18
0.6	11.1(2)	1.928(7)	-	2.3–40
1.0	2.51(9)	2.32(2)	-	2.3–40
1.5	2.7(3)	2.32(4)	11(2)	6.5–40
2.0	3.2(1)	2.3(1)	30(3)	6.5–40
2.5	2.6(1)	2.301(1)	79(4)	6.5–40
3.0	2.3(2)	2.30(1)	138(8)	6.5–40

**Figure 3:**
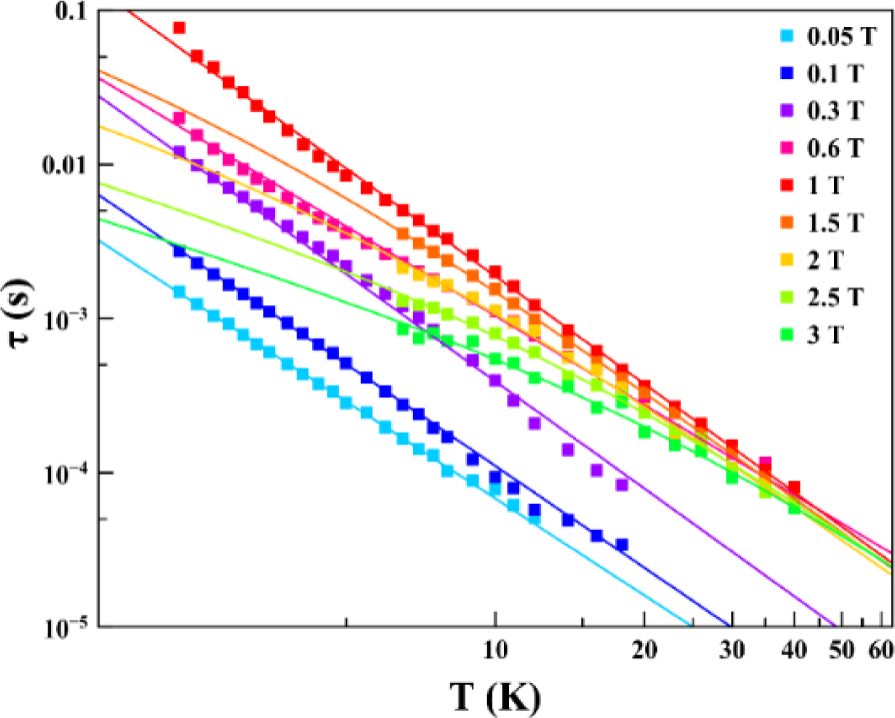
Temperature dependence of τ extracted from ac susceptibility measurements (see Figure S16).

The experimental values of τ as a function of the temperature at different magnetic fields are depicted in Figure 3. For all of the measured temperatures, τ increases with increasing magnetic field, reaches a maximum around 1 T, and then starts to decrease. This behavior of the relaxation rate with the magnetic field has two main contributions: at low fields, the relaxation is promoted by spin–spin and hyperfine interactions (inter- and intramolecular interactions), which are suppressed with increasing field. Instead, at higher fields, the relaxation is dominated by the spin–phonon direct mechanism (first term of eq 1) because more phonons with energy equal to the separation of the *m*_S_ states are present. The relaxation time of **1** shows its maximum at intermediate fields when the direct relaxation mechanism has not been activated, but the applied magnetic field is enough to suppress spin–spin and spin–nuclei interactions. As shown in Figures 4 and S16, this behavior is temperature independent.

**Figure 4:**
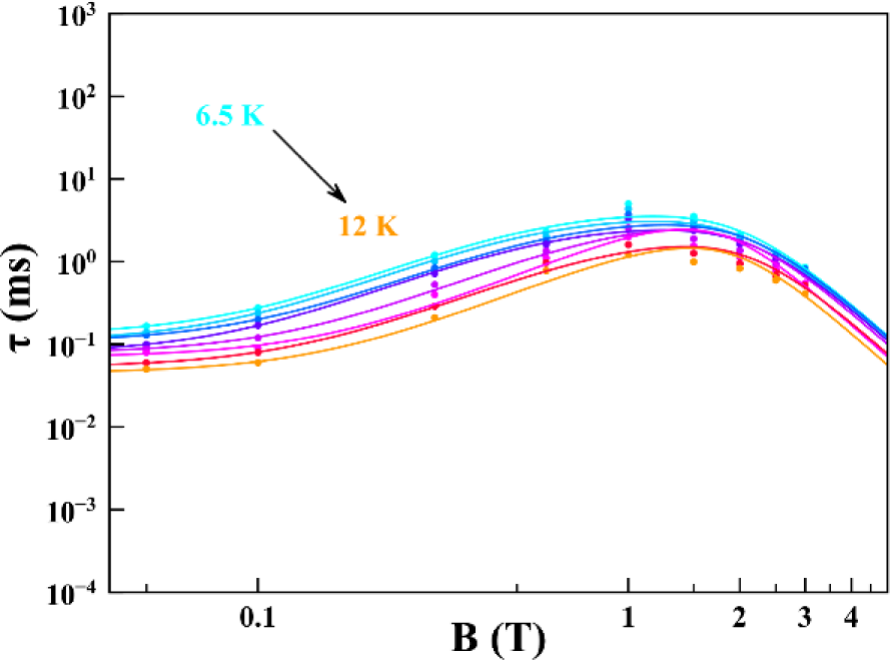
Dependence of τ with the magnetic field for **1** at different temperatures of up to 12 K. The solid line represents the best fit.

Considering these two different contributions of the magnetic field to the relaxation rate, the magnetic field dependence of τ (Table S5) has been fitted using the expression:

2 $$\tau^{-1}=cB^{4}+\left[d\left(1+eB^{2}\right)/\left(1+fB^{2}\right)\right]$$

where the first term is the field-dependent Raman (otherwise known as the Brons–Van Vleck equation), and the second term is the Direct pathway. The fitting curves at each temperature are depicted in Figures 4 and S16, and the best-fitting parameters are summarized in Table S6. The study of the dependence of τ with temperature under the application of different magnetic fields using the Debye model reveals that, from 0.05 to 1 T, a pure Raman relaxation mechanism occurs. From 1 T, participation of the direct process is needed, which is in good agreement with the fact that the direct mechanism should be more active at high fields (see above).

Among eq 2, especially interesting is the *d* parameter, which represents the zero-field relaxation and possesses an exponential dependence on temperature that is not related to an Orbach-like relaxation, rather it indicates that there are well-defined energy levels involved in spin–phonon relaxation.^56^ Given the nature of the relaxation processes taking place, these energy levels might correspond to the vibrational levels of the molecule. The value of the energy level at play can be determined through:

3 $$\ln\left(d\right)=\ln\left(a\right)-U_{eff}/K_{B}T$$

This relationship is confirmed in Figure S17 by representing ln­(*d*) vs 1/*T* yielding an energy barrier of 10.0(1) cm^–1^.

Furthermore, upon the application of the correlation:

4 $$U_{eff}=h\omega /2$$

We found that the obtained energy barrier comes from a vibrational mode of 20.0 cm^–1^. To further probe the presence of these low-energy phonon, we performed ultralow frequency Raman spectroscopy on a polycrystalline sample of 1 in the 0–100 cm^–1^ frequency range (THz region) at room temperature (Figure 5), where we found different overlapped absorptions between 10 and 30 cm^–1^ and a more intense peak at 40.0 cm^–1^ (Figures 5 and S18).

**Figure 5:**
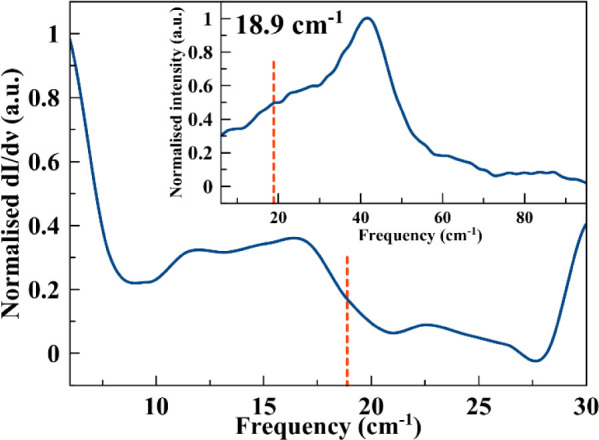
Derivative of the Raman spectra of **1** in the analyzed region. Inset: Raman spectra were in the 0–100 cm^–1^ range. The red dashed line marks the position of the correlated vibrational mode in both plots.

The expected vibrational mode calculated by applying the Brons–Van Vleck equation agreed with the possibility of having an absorption peak in the region of 15–30 cm^–1^, in particular with the absortion centered at 18.9 cm^–1^.

### EPR Characterization

Electron paramagnetic resonance (EPR) spectroscopy was used to probe the static magnetic properties and electron spin relaxation rates of **1**. The Q-band (ν_MW_ ≈ 34 GHz) electron spin echo (ESE)-detected EPR spectrum of a 0.5 mM frozen solution in a 3:1 dichloromethane:toluene mixture is shown in Figure 6.

**Figure 6:**
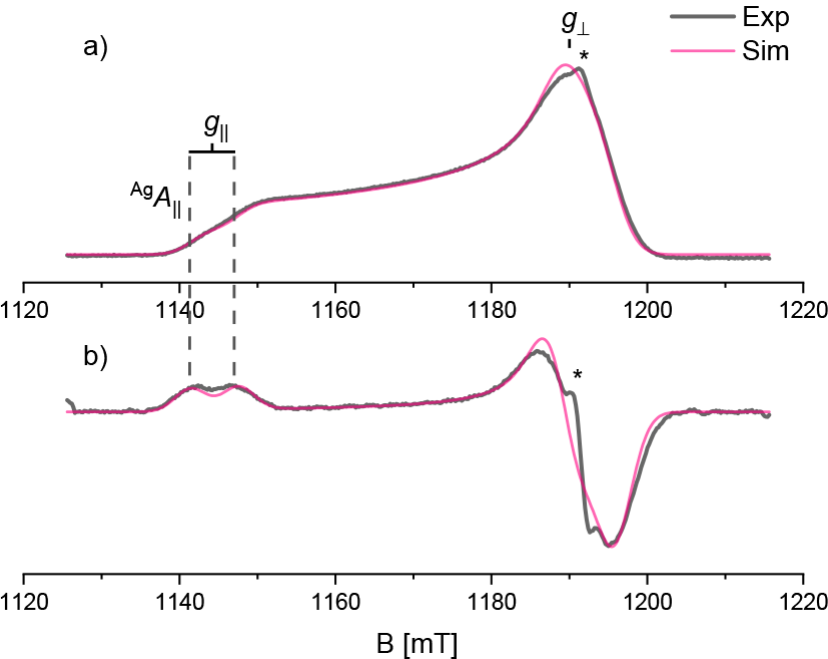
Q-band ESE-EPR spectrum recorded at a 40 K of 1. Panel b) shows the pseudomodulated absorption spectrum. The simulation is shown in magenta. The asterisk indicates a spurious radical signal. The resolved hyperfine splitting pattern due to ^107,109^Ag isotopes (*I* = 1/2) is indicated.

The spectrum was simulated assuming the spin-Hamiltonian in eq 5

5 $$Ĥ=\mu_{B}B\cdot \mathrm{g̿}\cdot Ŝ+Ŝ\cdot {\mathrm{A̿}}_{\mathrm{Ag}}\cdot {Î}_{\mathrm{Ag}}$$

with *g*_||,⊥_=[2.11, 2.025] and ^Ag^ *A*_||,⊥_=[168, 110] MHz (see Tables 2 and S7), whereby the value of the ^Ag^ *A*_⊥_ component is estimated based on the spectral linewidth. The values are in line with those reported in the literature for similar compounds.^57^

**Table 2:** Principal Values of ^Ag^ ***A***, ^N^ ***A*** and ^H^ ***A*** Tensors Derived from the Simulation of the Experimental Spectra^a^

	^107^Ag	^14^N(1)	^14^N(2)	^1^H
*A*_||_	168 ± 5	42 ± 2	47 ± 2	19 ± 0.5
*A*_⊥_	100 ± 10	33 ± 1	35; 36^b^ ± 2	6.0; 3.0^b^ ± 1

a All values are in units of MHz.

b The two values correspond to the *A*_*x*_ and *A*_*y*_ components of a rhombic tensor.

To precisely elucidate the bonding between the metal ion and its coordinating ligands, Electron–Electron DOuble Resonance (ELDOR)-detected NMR (EDNMR)^47^a,b was employed to measure the hyperfine coupling with the surrounding ligands (Figure 7). Compared to conventional Electron Nuclear DOuble Resonance (ENDOR), EDNMR offers higher sensitivity and avoids nucleus-dependent or pulse-dependent spectral artifacts.^48^

**Figure 7:**
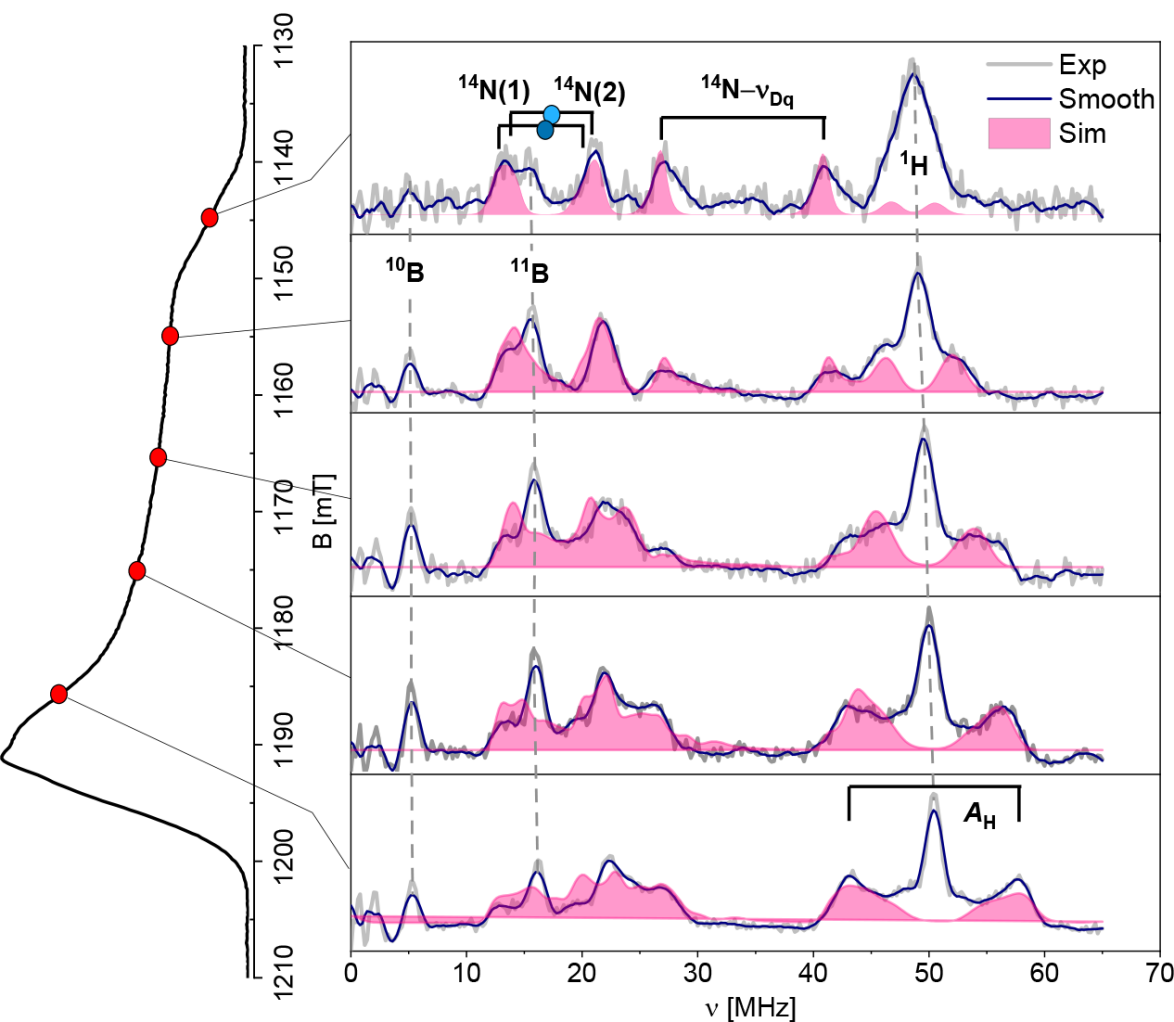
Orientation-selective Q-band EDNMR spectra after data point smoothing with the Savitzky–Golay filter (blue lines). Original data are shown as gray lines. The corresponding computer simulations are shown as pink shaded areas. The magnetic field setting at which each spectrum was recorded is indicated by the red dots on the echo-detected-EPR spectrum shown on the left panel. The EDNMR spectra were recorded at *T* = 20 K, using a Gaussian-shaped preparation pulse of duration *t*_HTA_= 9 μs and variable frequency. The detection pulse was a standard Hahn echo with *t*_π/2_ = 400 ns and τ = 600 ns. For clarity, the central line at zero frequency has been subtracted from the experimental spectra, and only the positive frequencies are plotted (the full spectra are shown in Figure S19).

In this experiment, selective microwave pulses simultaneously drive both electron paramagnetic resonance (EPR) and nuclear magnetic resonance (NMR) transitions within the spin manifold—so-called spin-forbidden transitions—where both electron and nuclear spin projections change (Δ*m*_S_ = ± 1, Δ*m*_I_ = ± 1, ±2, ... ). A typical EDNMR pulse sequence (Figure S18) consists of two microwave channels: one for detection and the other for selective excitation.

The detection channel employs a Hahn-echo sequence at a fixed frequency to monitor the intensity of an allowed EPR transition. The second microwave channel delivers a soft microwave pulse, termed a high-turning angle (HTA) pulse, whose frequency is typically swept symmetrically around the detection pulse frequency. When the HTA pulse frequency (ν_HTA_) matches a transition of the type (Δ*m*_S_ = ± 1, Δ*m*_I_ = ± 1, ± 2, ...), polarization transfer occurs, leading to a change in spin–echo intensity in the detection channel. As ν_HTA_ approaches the frequency of the detection pulses (ν_det_), the detected EPR transition saturates, causing the echo intensity to drop to zero at Δν_det_ = ν_HTA_ – ν_det_ = 0, a phenomenon known as the central blind spot.

By monitoring the echo intensity as a function of the difference between the two microwave frequencies (ν_HTA_ and ν_det_), the nuclear frequencies—corresponding to the hyperfine interaction of nuclei coupled to the electron spin—are detected. Weakly coupled ligand nuclei resonate at their respective Larmor frequency (ν*_I_*), with individual peaks split by the hyperfine coupling constant (*A*_I_). Strongly coupled nuclei resonate at *A*_I_/2, with peaks further split by 2ν_I_ to first order.

The theoretical treatment of EDNMR for an *S* = 1/2, *I* = 1 system is discussed in detail.^58,59^

Figure 7 presents orientationally selective EDNMR spectra recorded at different resonant magnetic fields. The spectrum obtained at the lowest magnetic field (top panel in Figure 7) corresponds to a single-crystal-like orientation, where only molecules with their *g*_||_ component aligned along the external magnetic field are excited. This spectrum exhibits two peaks at approximately ν ≈ 13.6 and ν ≈ 21.2 MHz, separated by approximately twice the ^14^N nuclear Larmor frequency (ν_N_ = 3.502 MHz at this field) and centered at ν ≈ 16.5 MHz. These peaks are attributed to strongly coupled nitrogens with a hyperfine coupling of *A ≈* 33 MHz.

Additionally, the double quantum (*Dq*) transitions associated with these strongly coupled nitrogen nuclei manifest as a second pair of peaks separated by 4ν_N_ and centered at 34.6 MHz (see labeling in Figure 7). At higher magnetic fields, the spectra become more complex and simulations indicate the presence of two distinct sets of nuclei (N(1) and N(2) in Figure 7), which nearly overlap at *g*_||_.

A prominent peak is observed at the proton Larmor frequency (ν_H_ ≈ 50 MHz), which is marked by a dashed line in Figure 7. This is mostly due to the magnetic interaction with the remote hydrogen nuclei. However, spectra recorded at higher magnetic fields show the onset of two transitions centered at ν_H_, which identify the presence of nearby protons (resonating in the weakly coupled regime, i.e., with resonances centered at the nuclear Larmor frequency and separated by the hyperfine coupling constant) with a maximum coupling of approximately 19 MHz at the largest magnetic field (bottom panel in Figure 7). Finally, inspection of the spectra shows the presence of two peaks centered at the Larmor frequency of the ^10^B and ^11^B isotopes (dashed lines in Figure 7).

A simulation analysis of the EDNMR spectra was performed by considering a four-spin system (*S* = 1/2, *I*_1_ = 1, *I*_2_ = 1, and *I* = 1/2) and neglecting the remote nuclei characterized by single resonance lines at the respective Larmor frequencies (^10,11^B and remote ^1^H). The result of the simulation at each magnetic field setting is shown in Figure 7 and confirms the presence of two different groups of N ligands (labeled as N(1) and N(2)), indicating a departure from ideal *D*_4h_ symmetry in the equatorial plane. This is also in agreement with the different distances of the two nitrogen ligand pairs, as measured from the X-ray structure (Figure 1). The full list of spin Hamiltonian parameters derived from the simulation is reported in Table S8, while the principal values of the hyperfine tensors of the relevant nuclei are listed in Table 2.

The hyperfine tensors can be decomposed in the isotropic (*a*_iso_) and anisotropic (*T*) components (see Section S5), from which the spin density repartition over the different atomic orbitals that contribute to the semi-occupied molecular orbital (SOMO) hosting the unpaired electron can be estimated. Taking into account that a 100% spin population in a ^107^Ag 5s and 4d orbital produces a hyperfine coupling constant of *a*_0_ = 1831 MHz and *b*_0_ = 58.5 MHz, respectively,^60^ the experimental values of *a*_iso_ = 123 MHz and *T* = 23 MHz imply a total spin population on the silver of ρ^Ag^ ≈ 45%, which is larger than the value (38%) reported for Ag­(TPP) complexes.^55^ The same treatment applied to the nitrogen hyperfine couplings (Section S5) provides insights into the s and p character of the ligand σ bond. Assuming the hyperfine coupling constants for unitary 2s and 2p N orbitals to be *a*_0_ = 1540.33 MHz and *b*_0_ = 50.9 MHz,^61^ the values ρ_s_ ≈2.3% and 2.5% and ρ_p_≈ 6.0% and 8.4% for N(1) and N(2) respectively are derived. Assuming that N(1) and N(2) correspond to pairs of equivalent nuclei, the overall spin density distribution over the four nitrogens is ρ^Ntot^ = 2­(ρ^N(1)^ + ρ^N(2)^) ≈ 38%. This value, summed with the 45% spin density estimated on Ag­(II), accounts for 83% of the spin density, which, considering the inherent approximations and the uncertainties in the determination of the ^Ag^ *A*_⊥_ component (Table 2), can be considered satisfactory.^62^ The lower spin density delocalization on the nitrogen ligands of **1**, with respect to the AgTPP complex, implies a smaller degree of covalency in the metal–ligand bond, which can be understood considering the different degrees of s–p hybridization in the two systems. The ^N^ρ_p_/ ^N^ρ_s_ ratio for **1** falls in the range 2.8–3.3 for N(1) and N(2), respectively, in agreement with the expected sp^3^ character of the CTH nitrogen ligands and is larger with respect to the value reported for the sp^2^ N ligands of AgTPP (^N^ρ_p_/ ^N^ρ_s_ = 2.3).^55^ The smaller p-character in the nitrogen sp^2^ hybrid orbital of the tetrapyrrole macroligand leads to a larger overlap with the metal orbitals, favoring spin delocalization over the ligand^63^ and explaining the larger ^14^N hyperfine couplings observed for the AgTPP complex (Table S7). This is also consistent with the difference in the Ag–N bonding distance for nitrogen ligands featuring different hybridizations. An average Ag–N distance of 2.092 Å is reported for sp^2^ TPP^64^ and pyridine ligands,^65^ which is to be compared to the average distance of 2.158 Å in (**1**).

A ^1^H hyperfine interaction, with a maximum coupling of about 19 MHz, is also observed in the EDNMR spectrum. Analysis of the proton hyperfine tensor indicates a metal–proton distance of the order of 0.24 nm. Besides providing direct experimental evidence of the spin density distribution in **1**, the EDNMR data establish its solution structure. Specifically, the detection of well-resolved and sharp hyperfine transitions for ^14^N demonstrate that **1** maintains a rigid equatorial coordination consistent with the short Ag–N bond distances derived from the diffraction studies, although the differences in the hyperfine coupling of the two sets of nitrogen ligands call for a distortion in the equatorial plane. The observation of ^10,11^B hyperfine couplings (^10^B and ^11^B with ν_I_ ≈ 5.4 and 16.2 MHz, respectively in Figure 7) reveals that even the poorly coordinating BF_4_^–^ anions are preserved in solution.

The spin relaxation properties were studied by temperature-dependent echo decay and inversion recovery experiments. The coherence (*T*_m_) and spin–lattice relaxation (*T*_1_) times in the temperature interval between 7 and 100 K were determined and are reported in Figure 8.

**Figure 8:**
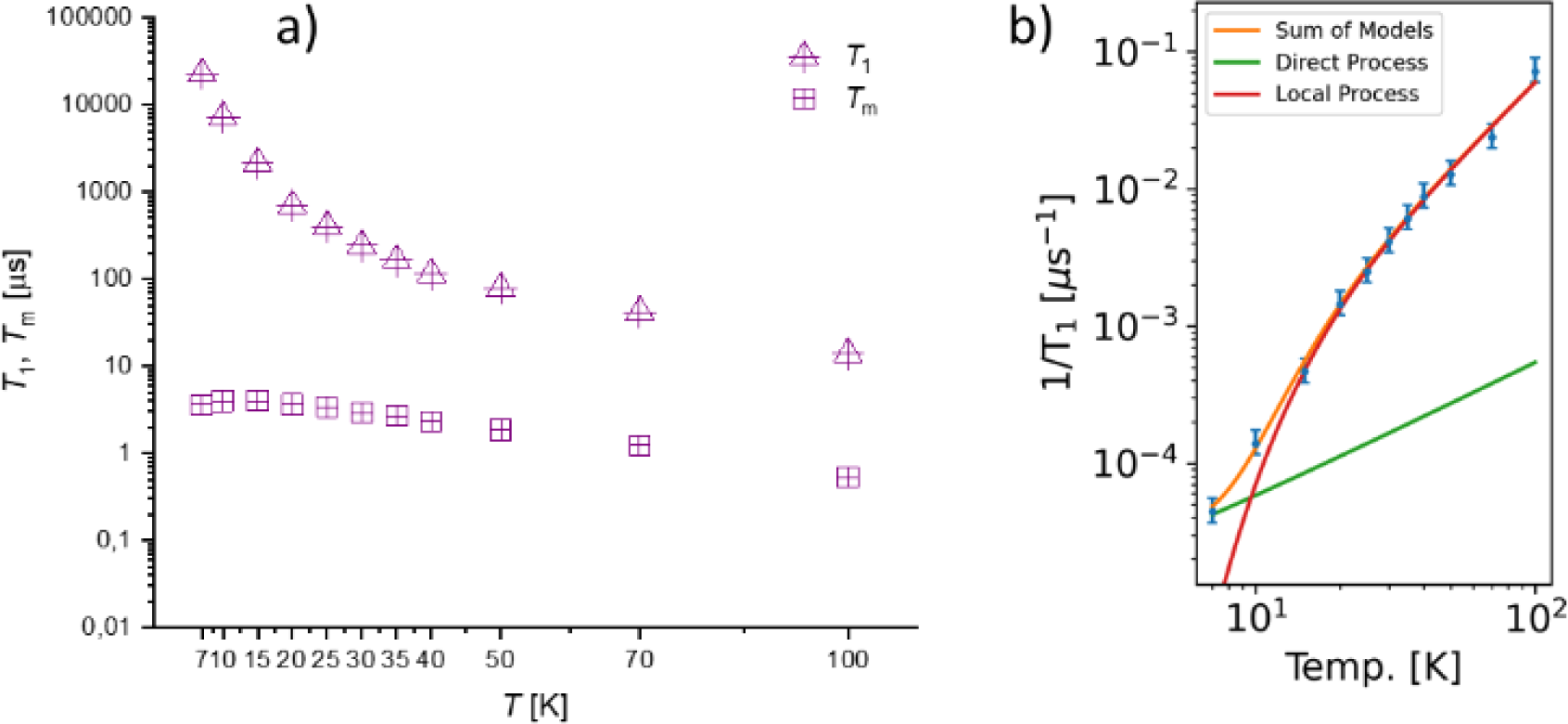
a) Temperature dependence of *T*_1_ (triangles) and *T*_m_ (squares). All experiments were measured at a magnetic field setting corresponding to the *g*_∥_ component of the EPR spectrum (B_0_ = 1149.7 mT). b) Fit of the relaxation rates based on eq 6. Details are given in the Supporting Information.

*T*_m_ values were extracted by fitting the experimental decay traces using a stretched exponential function. A *T*_m_ value of the order of 4 μs is recorded at 10 K, which drops below 1 μs at 100 K due to the increasing mobility of the solute as the glass softens (Figure 7). These values may be compared to those reported for silver­(II) tetratolylporphyrin (AgTTP) in glassy solution, where *T*_m_ values of 2.7 μs were measured at 15 K and X-band frequency.^43^ The values compare favorably suggesting that the coherence time is not significantly affected by the magnetic field (three times larger in our case) by the different degree of covalency in the metal–ligand bond or by the larger number of protons present in the CTH with respect to the porphyrin ligand.

The inversion recovery traces were fitted with a biexponential function, and the slow component is plotted in Figure 7 as a function of temperature. A *T*_1_ value of about 22 ms is observed at 10 K, consistent with the relaxation time extracted by temperature-dependent dc magnetic susceptibility measurements at 1 T (Figure S15). *T*_1_ decreases to about 14 μs at 100 K. No significant orientational dependence in both *T*_1_ and *T*_m_ was observed (Figure S20) probably due to the overlap of the Ag and N transitions, which blur out the orientation dependence even at Q-band frequency.^43^

The dependence of 1/*T*_1_ (ln scale) vs 1/*T* for **1** (Figure S21) shows a linear behavior in the high-temperature region (25–70 K), as observed for AgTTP.^43^ However, for **1,** a more general approach was used to model the temperature dependence of *T*_1_ over the whole temperature range. This was done by considering a direct mechanism of relaxation at low temperatures and a local vibrational mode responsible for the high-temperature relaxation, according to eq 6.

6 1T1=Adirexp(ℏωmw/kBT)exp(ℏωmw/kBT)−1+Alocexp(ℏωloc/kBT)(exp(ℏωloc/kBT)−1)2

In eq 6, the first term represents the direct mode ( $\omega_{\mathrm{mw}}$ is the Zeeman frequency, with $\omega_{\mathrm{mw}}/2\pi$ = 33.7 GHz), while the second term accounts for a Raman process promoted by an optical mode of frequency $\omega_{\mathrm{loc}}$ . This model fairly reproduces the temperature dependence of *T*_1_ providing the frequency of a potential low-frequency local vibrational mode with ℏω = 39.2 cm^–1^ (Table S5), in very good agreement with the mode at 40.0 cm^–1^ derived from ultralow-frequency Raman spectroscopy.

## Conclusions

The full characterization of the magnetic response of a novel macrocyclic derivative of the extremely unusual Ag^II^ paramagnetic cation has been performed by means of the combination of magnetic and spectroscopic techniques. It has been determined that the Ag^II^ spin carrier joins to the scarce family of *S* = 1/2 cations exhibiting slow relaxation of the magnetization and potential qubit performance. The combination of EDNMR and NMR spectroscopies confirms the maintenance of the structure in solution thanks to the detection of superhyperfine Ag–B and Ag–N (EDNMR) and Ag–F (NMR) interactions. Magnetometry measurements pointed out that the relaxation of magnetization occurs through the competition of two different relaxation paths: Direct and Raman. The Raman path has been studied through the Brons–Van Vleck equation, and it has been found that this occurs due to the presence of a vibronic barrier. Spin relaxation properties were studied through temperature-dependent echo decay and inversion recovery experiments to find the spin–lattice relaxation time and coherence time. The spin relaxation time of 22 ms extracted from EPR measurements is in good agreement with the value extracted using magnetometry techniques. The relaxation mechanisms were modeled considering direct and local modes relaxation, in good agreement with the magnetometry fittings. Comparison with the literature data reported for Ag­(II)­TTP complexes suggests that structural (ligand rigidity) and electronic (ligand hybridization and metal–ligand covalency) differences in the ligand scaffold do not significantly impact on the relaxation rates, which seem to be largely dictated by the metal ion (bearing in mind the different fields and frequencies used for the two compounds. While magnetometry measurements were able to correlate the low-frequency local vibrational mode with ℏω = 10 cm^–1^ with the Brons–Van Vleck equation, EPR measurements were able to find an additional vibrational mode with ℏω = 39.2 cm^–1^. Both modes were experimentally correlated with the Raman spectrum in the THz region. Syntheses of new Ag^II^ complexes with strictly square planar or tetrahedral environment are in due course to study the influence of the Ag^II^ environment on the SRM and decoherence processes.

## Supplementary Material



## Notes

Additional synthetic, structural, magnetic and spectroscopic data can be found in the Supporting Information (PDF)
