# Supplementary material for: Spin Coherence and Electron Spin Distribution of a Silver(II) S = 1/2 Molecular System
Source: Inorg Chem. 2025 Jun 11;64(28):14091–100. doi: 10.1021/acs.inorgchem.5c00203 (PMC12284864; doi:10.1021/acs.inorgchem.5c00203)
Supplement: Supplementary file 1 [file ic5c00203_si_001.pdf]

Supporting Information

Spin coherence and electron spin distribution of a Silver(II) *S* = ½ molecular system

Joan Serra,[a] Enrico Salvadori [b], Yu-Kai Liao, [b] Albert Gallén,[a] Albert Escuer, [a] Mario Chiesa[b] and Júlia Mayans*[a,c]

[a] Departament de Química Inorgànica i Orgànica, Secció de Química Inorgànica, Universitat de Barcelona, Marti i Franques 1-11, Barcelona-08028, Spain.

[b] Department of Chemistry and NIS Centre, University of Torino, Via Giuria 7, Torino 10125, Italy

[c] Institut de Nanociència i Nanotecnologia (IN2UB), Universitat de Barcelona, Barcelona-08028, Spain.

Table of contents

1- Characterization

Figure S1. IR spectrum for complex [Ag(*m*-CTH)(BF_4)$_2$].
Figure S2. Comparison of the normalized UV-VIS absorption spectra of **1** with the ClO_4^- analogue.
Figure S3. ^{1}H-NMR (400 MHz, $CDCl_3$, 298 K) of CTH and assignment (bottom).
Figure S4. ^{1}H-NMR (400 MHz, $CDCl_3$, 298 K) of **1.**
Figure S5. Stacked ^{1}H-NMR (400 MHz, $CDCl_3$, 298 K) of CTH (top) and **1** (bottom).
Figure S6. ^{1}H-^{1}H COSY 2D NMR (400 MHz, $CDCl_3$, 298 K) of **1**.
Figure S7. ^{19}F-NMR (376.5 MHz, $CDCl_3$, 298 K) of **1**.

2- Structural information

Table S1. Crystal data and refinement details for complex **1**.
Table S2. Selected bond lengths (Å) and angles (º) for **1**.
Figure S8. Packing of the structure of [Ag(*m*-CTH)(BF_4)$_2$] viewed along a axis.
Figure S9. Coordination environment for the Ag^{II} cation in complex **1** compared with the ideal octahedral geometry.

3- DC and AC data

Figure S10. Field dependence of magnetization of 1 at 2 K. Inset: Temperature dependence of $\chi_M T$ of 1.
Figure S11. Temperature dependence of the real and imaginary components of the magnetic susceptibility for 1 as a function of the applied magnetic field at a fixed frequency of 1000 Hz.
Figure S12. Frequency dependence of χ' and χ'' of compound **1**.
Figure S13. Cole-Cole plots of compound **1** at the measured fields.
Table S3. Tau vs. temperature values for compound **1** at the measured fields.
Figure S14. Logarithmic temperature dependence of the relaxation time for compound **1** under the application of different magnetic fields.
Table S4. Slopes at different fields at low and high temperatures.
Figure S15. Fitted plots of the temperature dependence of the relaxation times for compound **1** as a function of the applied magnetic field.
Figure S16. Magnetic field dependence of τ^{-1} extracted from ac susceptibility measurements for compound **1** at different temperatures.
Table S6. Best-fit parameters of the model used to reproduce the field dependence of the magnetization relaxation for **1**.
Figure S17. Arrhenius plot of d parameters extracted from the fit of the Brons - van Vleck model.

4- EPR

Figure S18. EDNMR pulse sequence and energy levels diagram of an *S* = 1/2 and *I* = 1 electron−nuclear system.
Figure S19. Experimental EDNMR spectra at Q-band frequency and *T* = 20.

Table S7. Spin Hamiltonian parameters for silver (II) nitrogen complexes.
Table S8. Spin-Hamiltonian parameters used for the simulation of the EDNMR spectra.

5- ^{14}N Hyperfine coupling and determination of spin density over the coordinating nitrogen ligands:

Figure S20. Resonant field dependence of T_1 and Tm at 40K.
Table S9. Best fit parameters obtained using **Equation 1** of main text to reproduce the T dependence of T_1 for compound 1.
Figure S21. Linear plot of $1/T_1$ (ln scale) against $1/T$.

1- Characterization.

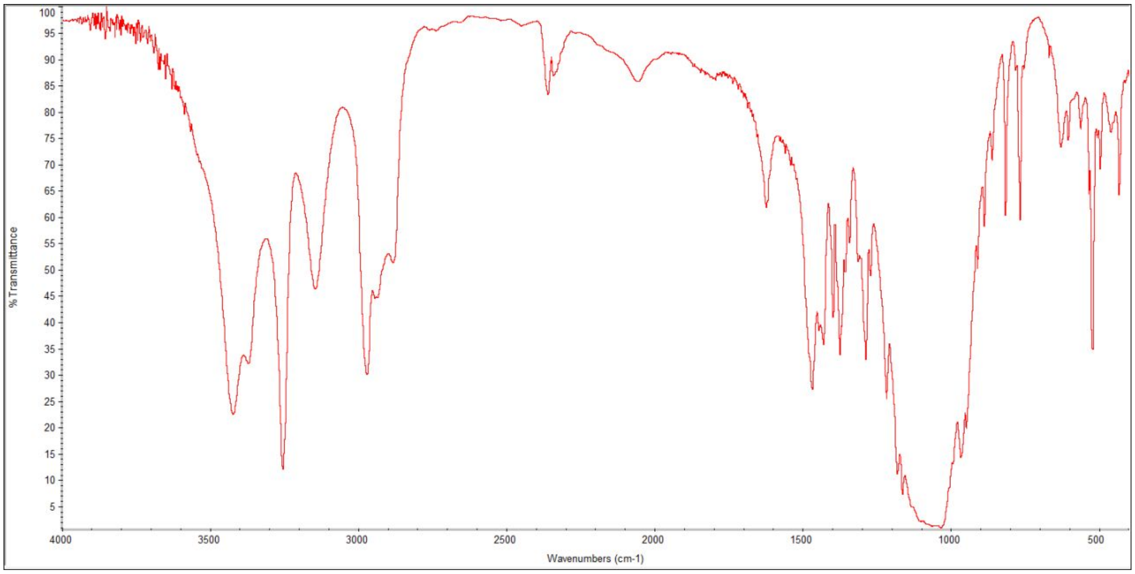

Figure S1. IR spectrum for complex [Ag(*m*-CTH)(BF_4)$_2$]. Stretching N-H, 3423-3255; stretching aliphatic C-H, 2971-2843; stretching B-F, 1034; bending BF_4^-, 521 cm^{-1}.

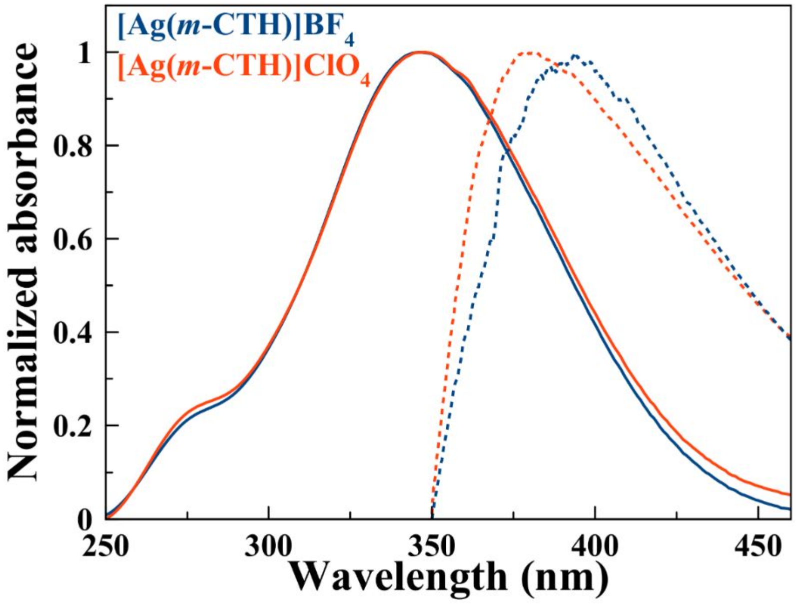

Figure S2. Comparison of the normalized UV-VIS absorption spectra of **1** with the ClO_4^- analogue in a methanolic solution (continuous line) and in solid diluted in a KBr pellet (dashed line).

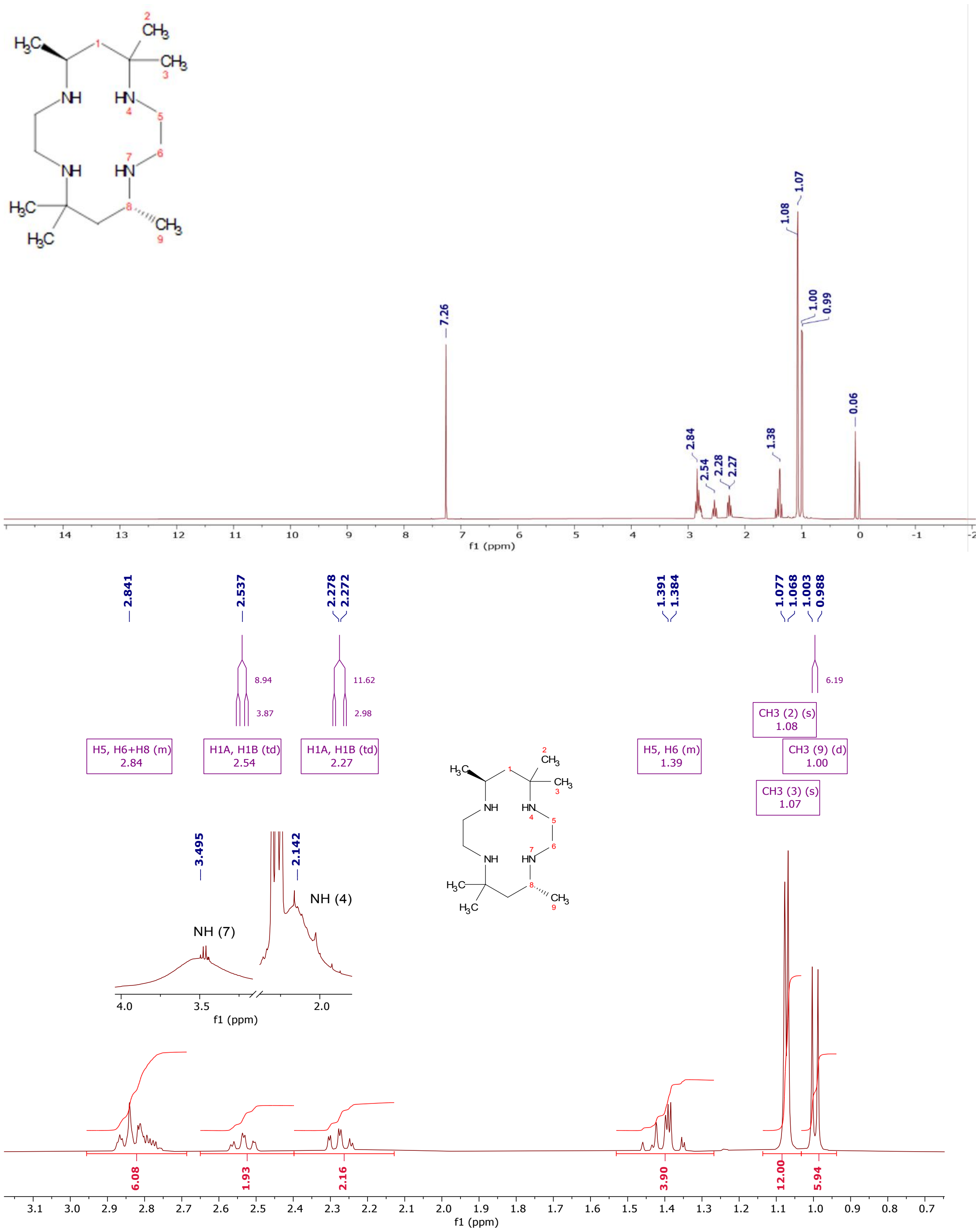

Figure S3. Top, ^{1}H-NMR (400 MHz, $CDCl_3$, 298 K) of CTH and assignment (bottom). δ (ppm) 3.50 (br.s, 2H, NH), 2.90 – 2.73 (m, 6H, CH_2, CH), 2.54 (td, $^{2}J_{H-H}$ = 8.9 Hz, $^{3}J_{H-H}$ = 3.9 Hz, 2H, CH_2), 2.27 (td, $^{2}J_{H-H}$ = 11.6 Hz, $^{3}J_{H-H}$ = 3.0 Hz, 2H, CH_2), 2.14 (br.s, 2H, NH), 1.59 – 1.28 (m, 4H, CH_2), 1.08 (s, 6H, CH_3), 1.07 (s, 6H, CH_3), 1.00 (d, J = 6.2 Hz, 6H, CH_3).

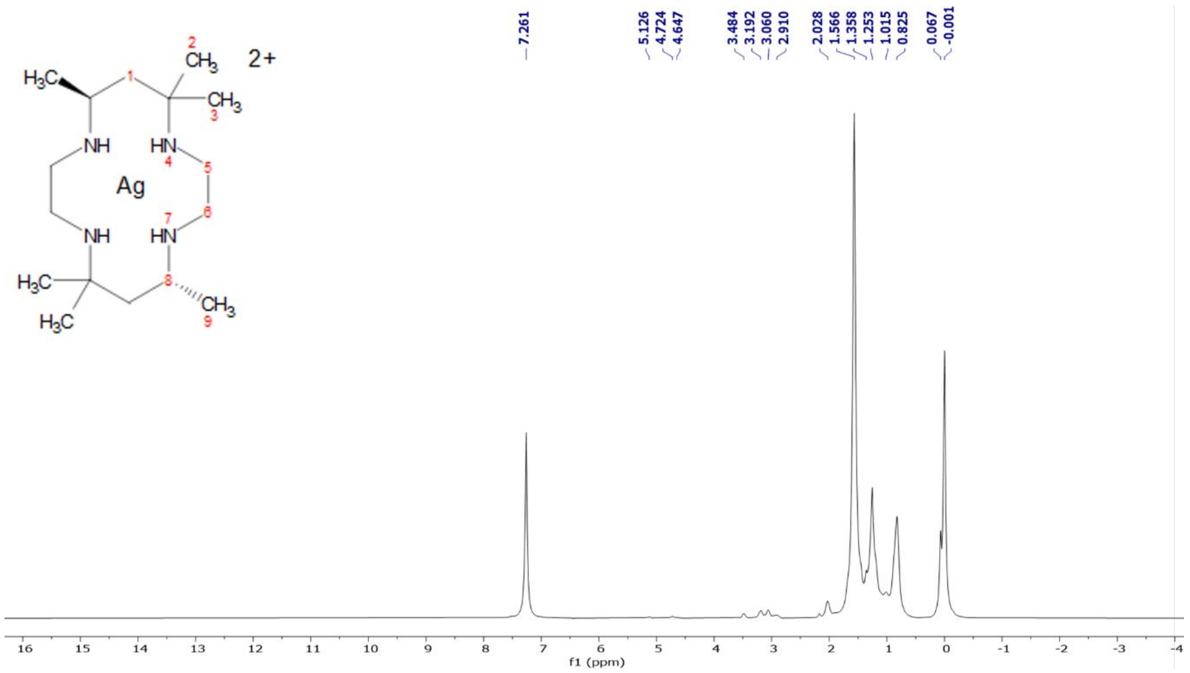

Figure S4. ^{1}H-NMR (400 MHz, $CDCl_3$, 298 K) of **1.** δ (ppm) 3.48 (d, $^{2}J_{H-H}$ = 7.4 Hz, 1H), 3.19 (d, $^{2}J_{H-H}$ = 12.0 Hz, 2H), 3.06 (br.s, 2H), 2.91 (br.s, 1H), 2.17 (br.s, 1H), 2.02 (br.s, 4H), 1.58 (br.s, 14H), 1.25 (br.s, 6H), 0.83 (br.s, 2H).

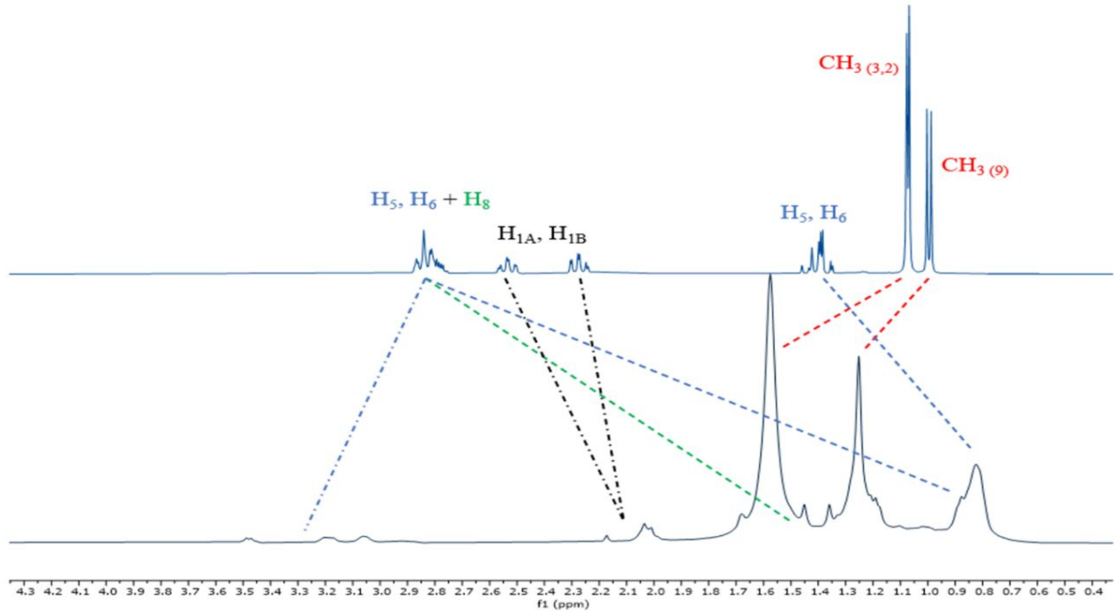

Figure S5. Stacked ^{1}H-NMR (400 MHz, $CDCl_3$, 298 K) of CTH (top) and **1** (bottom).

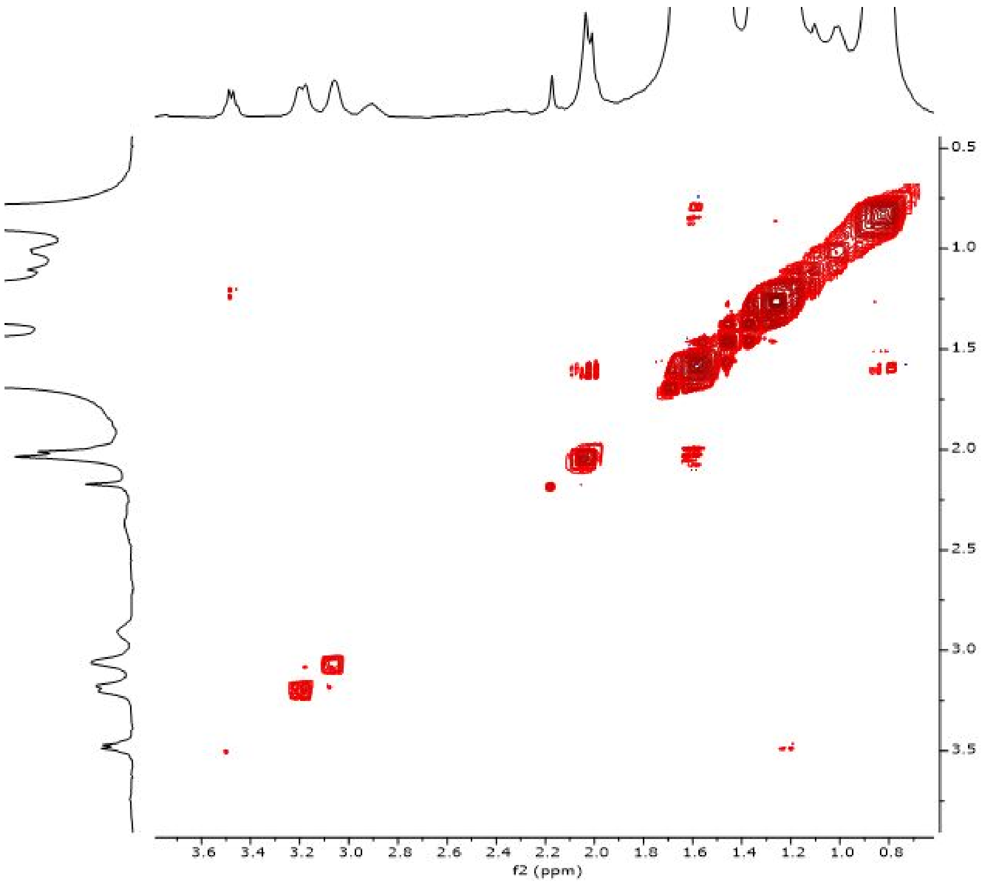

Figure S6. ^{1}H-^{1}H COSY 2D NMR (400 MHz, $CDCl_3$, 298 K) of **1**.

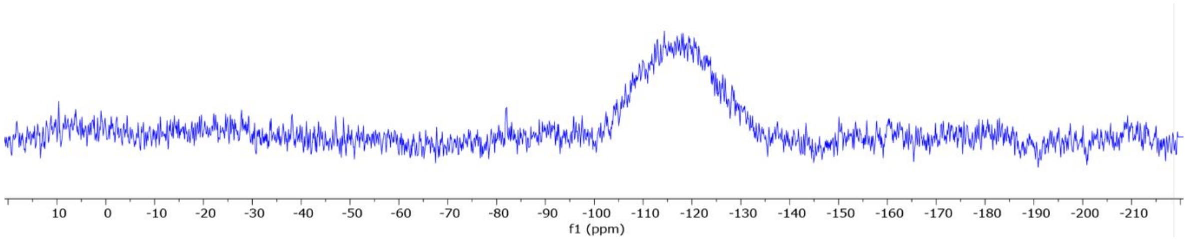

Figure S7. ^{19}F-NMR (376.5 MHz, $CDCl_3$, 298 K) of **1**, δ (ppm) -118.2 (br s, BF_4^-).

2- Structural information.

Table S1. Crystal data and refinement details for complex 1.

	**1**
Formula	$C_{16}H_{36}AgB_2F_8N_4$
FW	565.98
System	Monoclinic
Space group	C 2/c
a/Å	13.5344(14)
b/Å	11.4765(12)
c/Å	15.943(2)
α/deg.	90
β/deg.	112.839(4)
γ/deg.	90
V/Å3	2282.3(5)
Z	4
T, K	100(2)
λ(MoKα), Å	0.71073
ρ_{calc}, g□cm^{-3}	1.647
μ(MoKα), mm^{-1}	0.959
F (000)	1156
R	0.0284
ωR^2	0.0893

Table S2. Selected bond lengths (Å) and angles (º) for complex 1.

Ag1-N1	2.160(1)	F1-Ag-N1	78.04(4)
Ag1-N2	2.156(2)	F1-Ag-N2	79.06(5)
Ag1-F1	2.879(1)	F1-Ag-N1’	101.93(4)
N1-Ag-N2	84.40(5)	F1-Ag-N2’	100.94(5)
N1-Ag-N2’	95.60(5)		

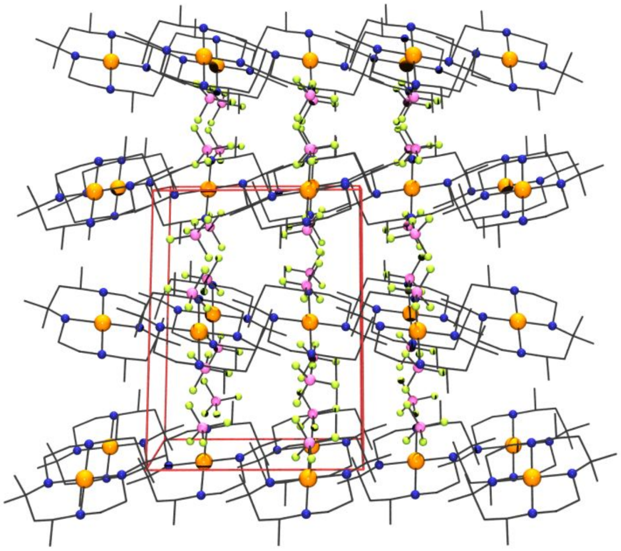

Figure S8. Packing of the structure of [Ag(*m*-CTH)(BF_4)$_2$] viewed along *a* axis. Color code: orange, Ag; navy, N; green, F; pink, B; black, C; red, cell axis. H atoms have been removed for clarity.

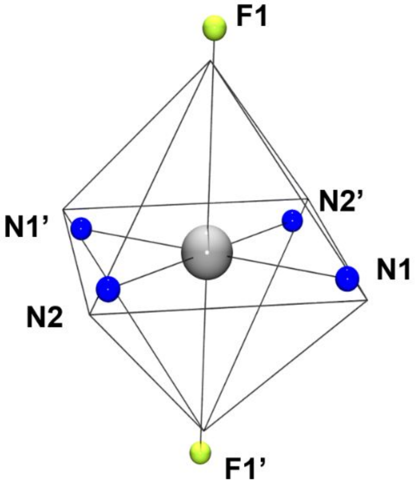

Figure S9. Coordination environment for the Ag^{II} cation in complex **1** compared with the ideal octahedral geometry. SHAPE2[1] analysis indicate a deviation coefficient from the regular octahedron of 3.37, derived from the Jahn-Teller distortion.

3- DC and AC data.

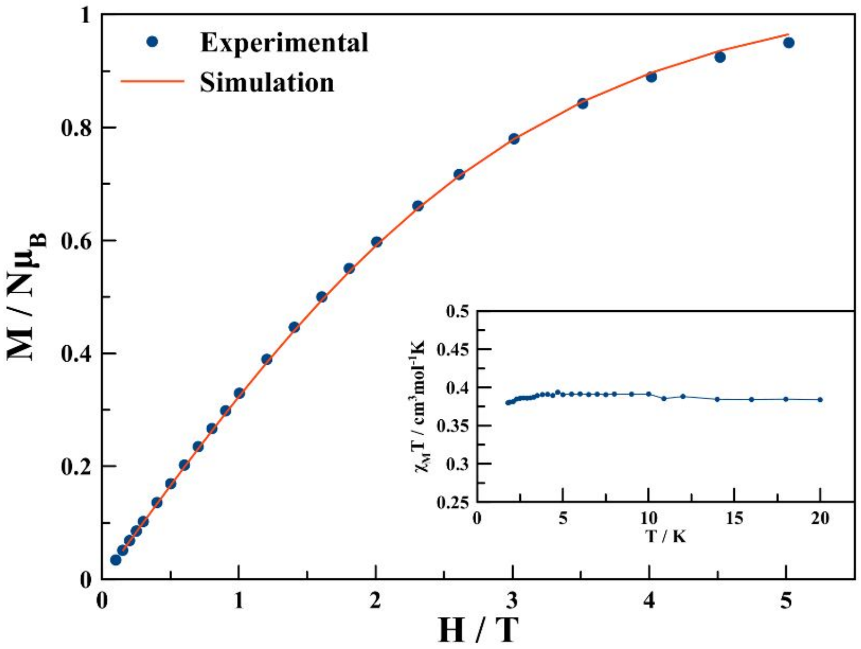

Figure S10. Field dependence of magnetization of **1** at 2 K. Inset: Temperature dependence of $\chi_M T$ of **1**.

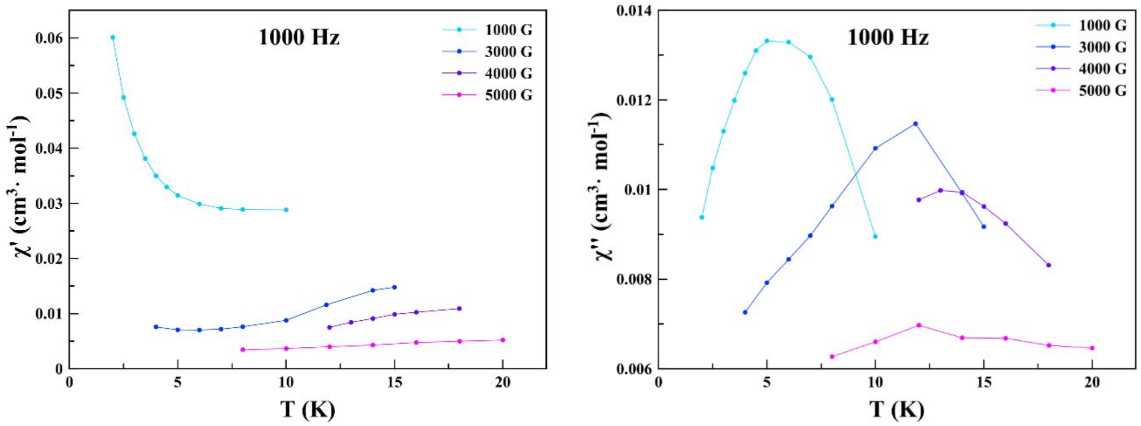

Figure S11. Temperature dependence of the real (left) and imaginary (right) component of the magnetic susceptibility for **1** as a function of the applied magnetic field at a fixed frequency of 1000 Hz.

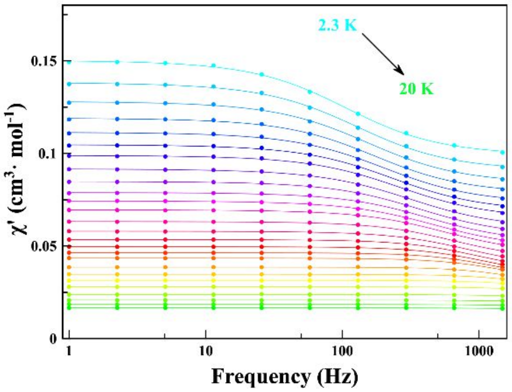
2.3 K
20 K
χ' (cm³· mol⁻¹)
Frequency (Hz)

0.05 T

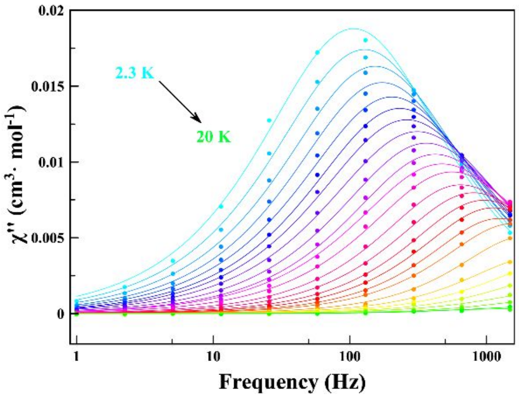
2.3 K
20 K
χ'' (cm³· mol⁻¹)
Frequency (Hz)

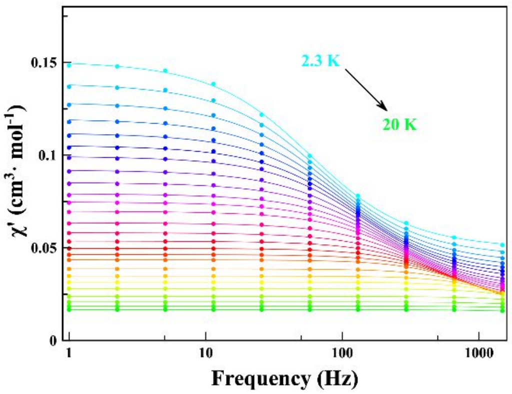
2.3 K
20 K
χ' (cm³· mol⁻¹)
Frequency (Hz)

0.1 T

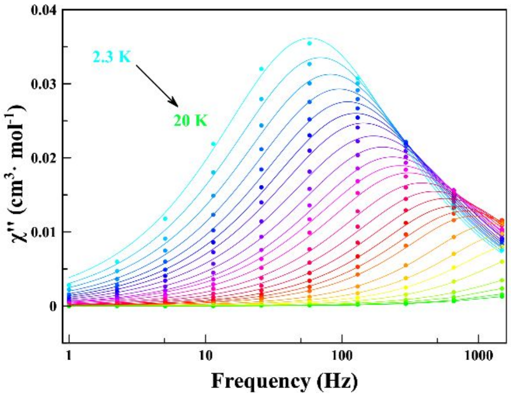
2.3 K
20 K
χ'' (cm³· mol⁻¹)
Frequency (Hz)

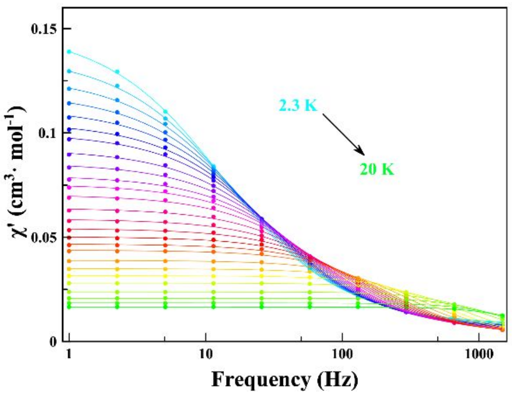
2.3 K
20 K
χ' (cm³· mol⁻¹)
Frequency (Hz)

0.3 T

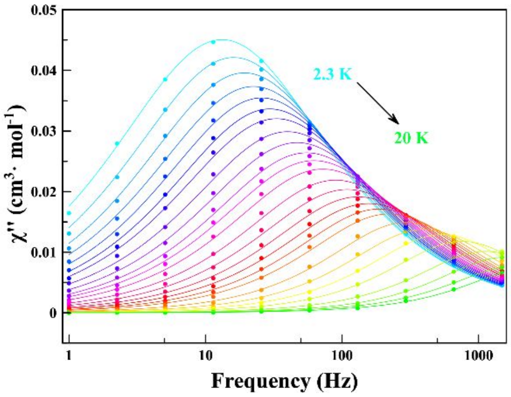
2.3 K
20 K
χ'' (cm³· mol⁻¹)
Frequency (Hz)

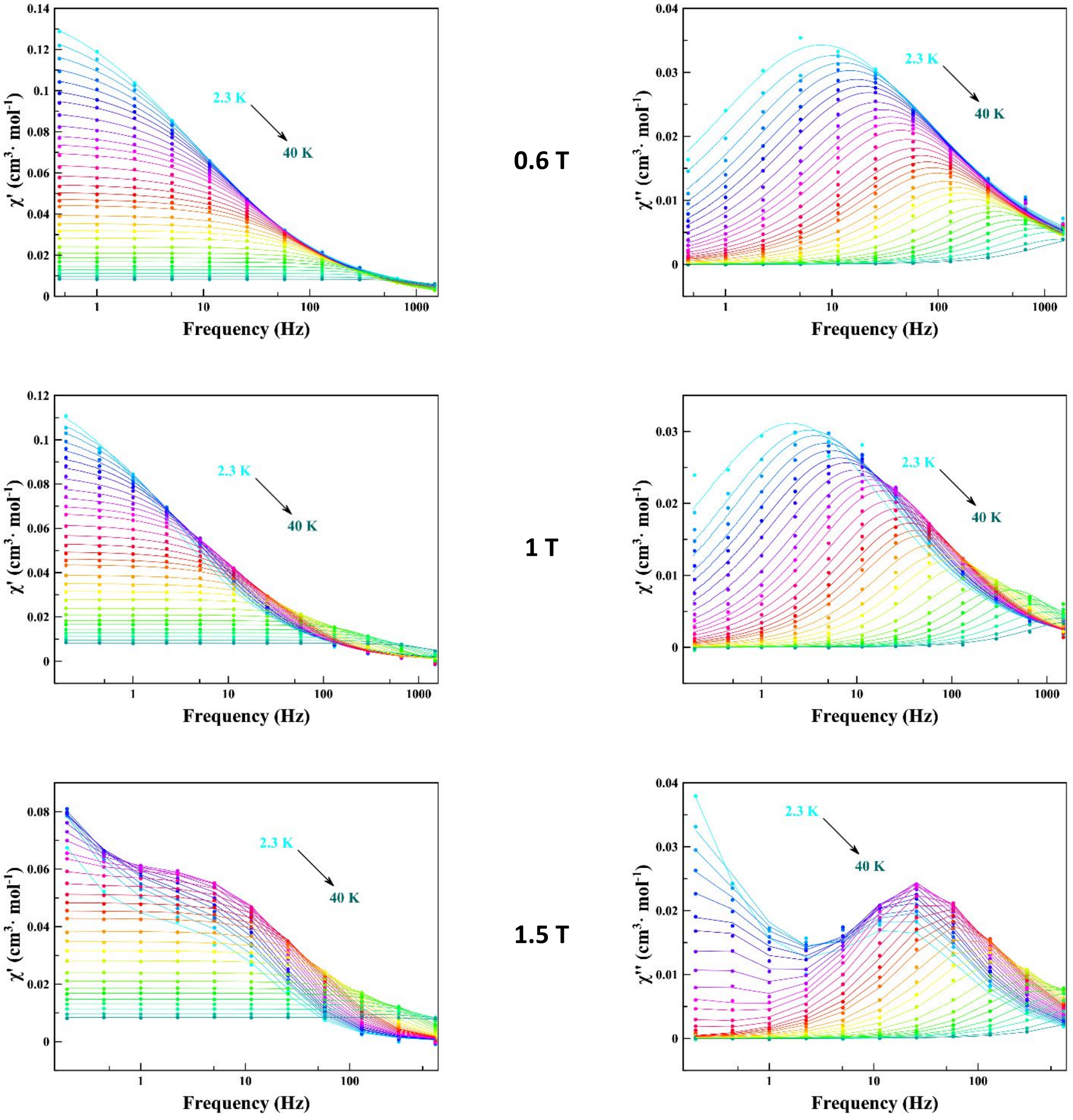
0.6 T
1 T
1.5 T
2.3 K
40 K
χ' (cm³· mol⁻¹)
χ'' (cm³· mol⁻¹)
Frequency (Hz)

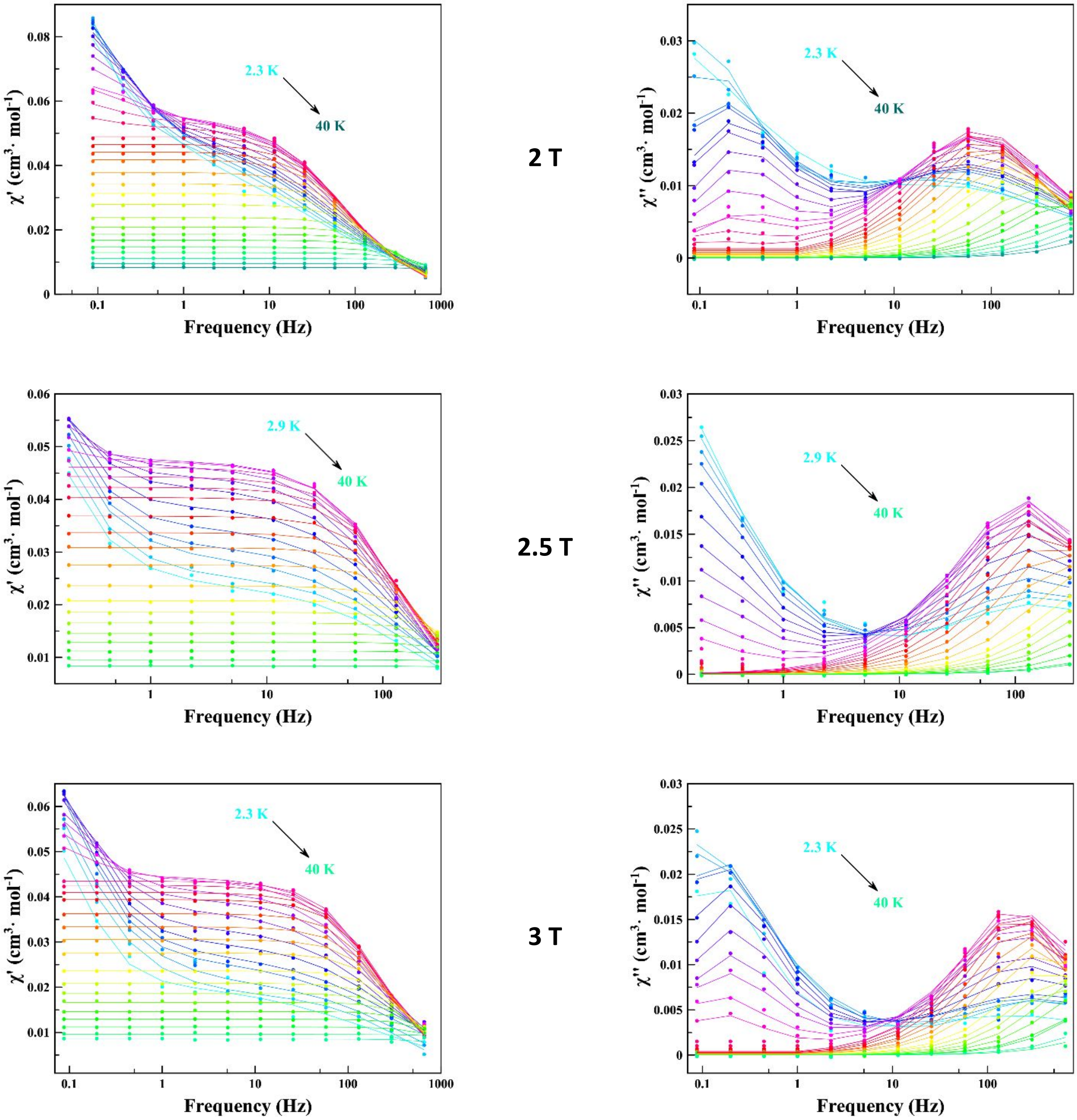

Figure S12. Frequency dependence of χ' (left column) and χ'' (right column) of compound **1** at 0.05, 0.1, 0.3, 0.6, 1.0, 1.5, 2.0, 2.5 and 3.0 T. Solid lines show the best fit of the plots.

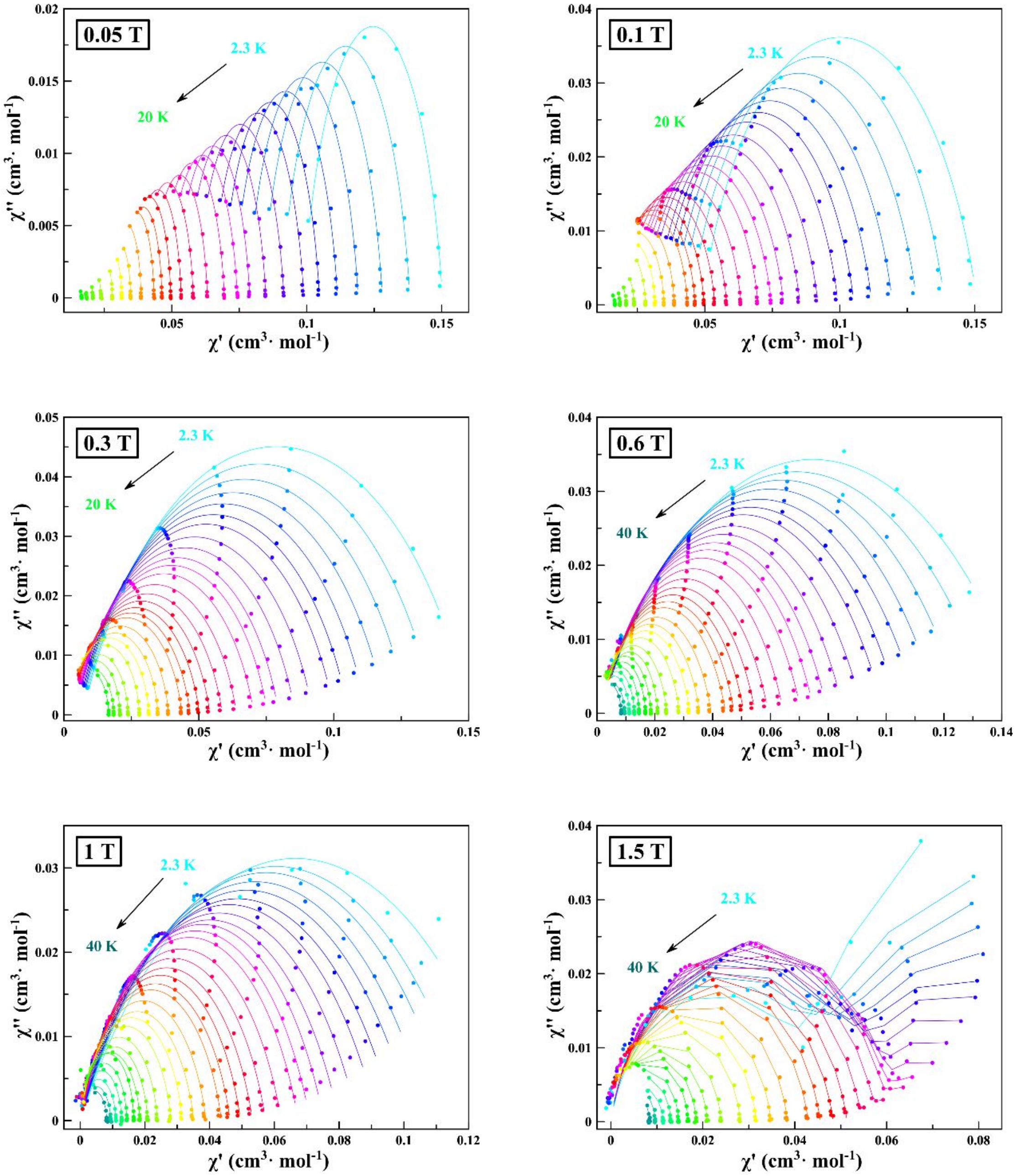
0.05 T
2.3 K
20 K
0.1 T
2.3 K
20 K
0.3 T
2.3 K
20 K
0.6 T
2.3 K
40 K
1 T
2.3 K
40 K
1.5 T
2.3 K
40 K
χ'' (cm³· mol⁻¹)
χ' (cm³· mol⁻¹)

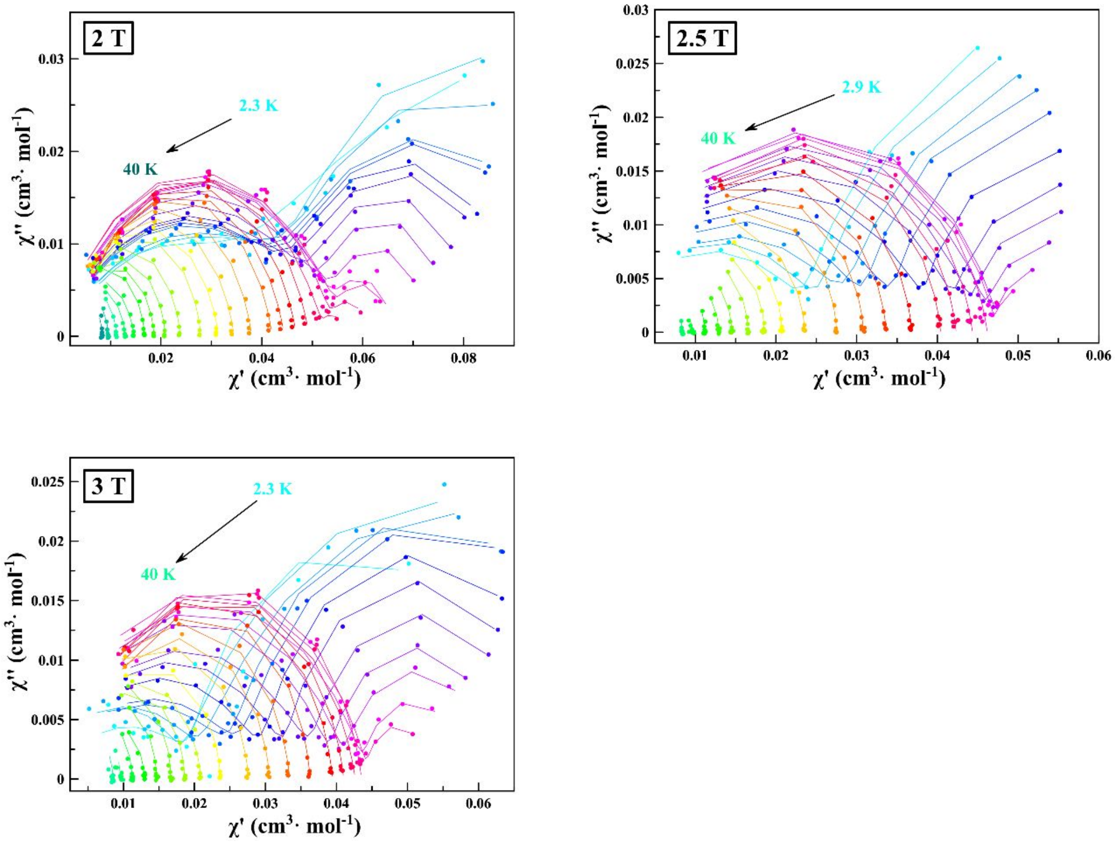

Figure S13. Cole-Cole plots of compound 1 at 0.05, 0.1, 0.3, 0.6, 1.0, 1.5, 2.0, 2.5 and 3.0 T. Solid lines show the best fit of the plots.

Table S3. Tau vs. temperature values for compound **1** at the measured fields.

**0.05 T**		**0.1 T**	
**T (K)**	**τ (s)**	**T (K)**	**τ (s)**
2.30119	0.001491	2.30119	0.002733
2.50051	0.00124	2.50051	0.002287
2.70037	0.001039	2.70037	0.001934
2.89987	9.22E-04	2.89987	0.001666
3.10016	7.84E-04	3.10016	0.001442
3.30008	6.86E-04	3.30008	0.001263
3.4999	6.07E-04	3.4999	0.001109
3.79992	5.09E-04	3.79992	9.34E-04
4.0921	4.38E-04	4.0921	8.01E-04
4.39951	3.77E-04	4.39951	6.80E-04
4.69758	3.36E-04	4.69758	5.97E-04
4.99728	2.84E-04	4.99728	5.15E-04
5.49793	2.46E-04	5.49793	4.15E-04
5.99676	1.98E-04	5.99676	3.38E-04
6.49791	1.66E-04	6.49791	2.77E-04
6.99605	1.42E-04	6.99605	2.40E-04
7.4958	1.30E-04	7.4958	1.96E-04
7.99396	1.03E-04	7.99396	1.71E-04
8.99461	8.89E-05	8.99461	1.22E-04
9.99668	7.84E-05	9.99668	9.40E-05
10.88828	6.17E-05	10.88828	7.98E-05
12.00078	5.11E-05	12.00078	5.74E-05
		13.99801	4.92E-05
		15.99953	3.90E-05
		17.99997	3.41E-05
**0.3 T**		**0.6 T**	
**T (K)**	**τ (s)**	**T (K)**	**τ (s)**
2.30119	0.012024	2.30038	0.020067
2.50051	0.009883	2.50047	0.01557
2.70037	0.008247	2.69988	0.012601
2.89987	0.007081	2.8996	0.010762
3.10016	0.006162	3.09922	0.009286
3.30008	0.005368	3.29874	0.008079
3.4999	0.004793	3.49963	0.007185
3.79992	0.003984	3.79906	0.006061
4.0921	0.003382	4.10181	0.005202

4.39951	0.002885	4.39774	0.004496
4.69758	0.002551	4.69771	0.004053
4.99728	0.002196	5.00028	0.003595
5.49793	0.00177	5.50024	0.00308
5.99676	0.001451	5.99992	0.002621
6.49791	0.001209	6.50038	0.002306
6.99605	0.001012	7.00038	0.002021
7.4958	8.43E-04	7.50021	0.001805
7.99396	7.15E-04	8.00051	0.001633
8.99461	5.35E-04	8.99967	0.001346
9.99668	3.99E-04	9.99975	0.001115
10.88828	2.93E-04	10.88987	9.67E-04
12.00078	2.10E-04	11.99916	7.77E-04
13.99801	1.41E-04	14.0004	5.68E-04
15.99953	1.04E-04	16.00009	4.68E-04
17.99997	8.33E-05	18.00009	3.45E-04
		20.00023	2.82E-04
		22.99932	2.44E-04
		26.00016	1.73E-04
		29.99983	1.33E-04
		35.00009	1.16E-04
		39.99987	6.82E-05
**1 T**		**1.5 T**	
**T (K)**	**τ (s)**	**T (K)**	**τ (s)**
2.30067	0.07699	6.5	0.003538
2.5	0.050375	7	0.003082
2.7	0.04267	7.50031	0.002705
2.90001	0.034072	8.00028	0.002392
3.0998	0.029378	8.99996	0.001892
3.29991	0.024152	9.99993	0.00155
3.49988	0.020432	10.89123	0.001258
3.80237	0.016645	11.99953	9.84E-04
4.10094	0.013387	13.99969	7.03E-04
4.39812	0.011317	15.9999	5.20E-04
4.69965	0.00971	18.00021	4.08E-04
5.00021	0.008446	20.00038	3.32E-04
5.50095	0.007027	22.99993	2.44E-04
6.00003	0.005888	26.00068	1.80E-04
6.49989	0.005036	30.00001	1.34E-04
6.99992	0.004339	34.99961	9.39E-05
7.49986	0.003724	40.00081	6.73E-05

7.9997	0.003299		
8.99892	0.002581		
10.0004	0.002006		
10.891	0.001614		
11.9996	0.001218		
14.0003	8.38E-04		
16.0003	6.13E-04		
18.0001	4.65E-04		
20.0001	3.67E-04		
22.9999	2.67E-04		
26.0004	2.08E-04		
29.9995	1.51E-04		
35.0001	1.05E-04		
40.0005	8.09E-05		
**2 T**		**2.5 T**	
**T (K)**	**τ (s)**	**T (K)**	**τ (s)**
6.5	0.002134	6.5	0.0013
7	0.001928	7	0.001228
7.50031	0.001743	7.50031	0.001179
8.00028	0.001645	8.00028	0.00107
8.99996	0.001366	8.99996	9.40E-04
9.99993	0.001127	9.99993	8.03E-04
10.89123	9.48E-04	10.89123	6.94E-04
11.99953	8.33E-04	11.99953	6.04E-04
13.99969	5.61E-04	13.99969	4.22E-04
15.9999	4.65E-04	15.9999	3.70E-04
18.00021	3.56E-04	18.00021	3.15E-04
20.00038	2.60E-04	20.00038	2.46E-04
22.99993	1.70E-04	22.99993	1.91E-04
26.00068	1.38E-04	26.00068	1.66E-04
30.00001	1.07E-04	30.00001	1.01E-04
34.99961	8.49E-05	34.99961	7.53E-05
40.00081	6.19E-05	40.00081	6.68E-05
**3 T**			
**T (K)**	**τ (s)**		
6.5	8.53E-04		
7	7.47E-04		
7.50031	8.05E-04		
8.00028	7.09E-04		
8.99996	7.11E-04		

9.99993	5.47E-04
10.89123	5.17E-04
11.99953	4.11E-04
13.99969	3.63E-04
15.9999	2.64E-04
18.00021	2.86E-04
20.00038	1.83E-04
22.99993	1.52E-04
26.00068	1.38E-04
30.00001	9.25E-05
40.00081	5.85E-05

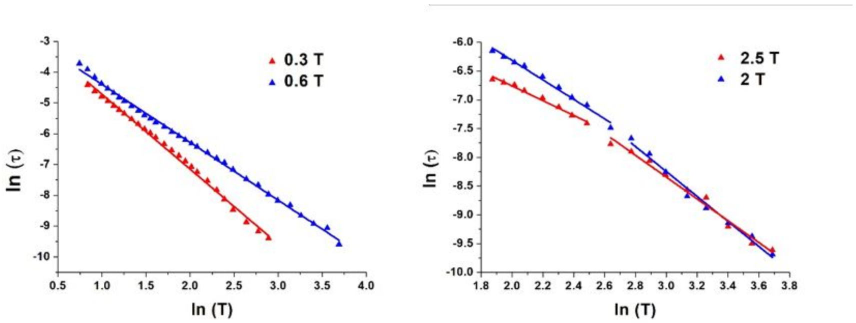

Figure S14. Logarithmic temperature dependence of the relaxation time for compound **1** under the application of different magnetic fields, indicating a different dependence of the relaxation time at low and high temperature regions at 2 and 2.5 T (right) as a consequence of the different dominancy of the relaxation mechanisms involved in. Such behavior is not observed at 0.6 T since there is only one relaxation mechanism involved.

Table S4. Slopes at 0.3 T (2.3-18 K), 0.6 T (6.5-40 K), 2 T (6.5-14 K and 14-40 K) and 2.5 T (6.5-12 K and 14-40 K).

**Magnetic field**	**Slope**	
0.3 T	-2.43 (R = 0.997)	
0.6 T	-1.88 (R = 0.996)	
	**Low temperature region**	**High temperature region**
2 T	-1.69 (R = 0.985)	-2.17 (R = 0.987)
2 .5 T	-1.28 (R = 0.983)	-1.89 (R = 0.983)

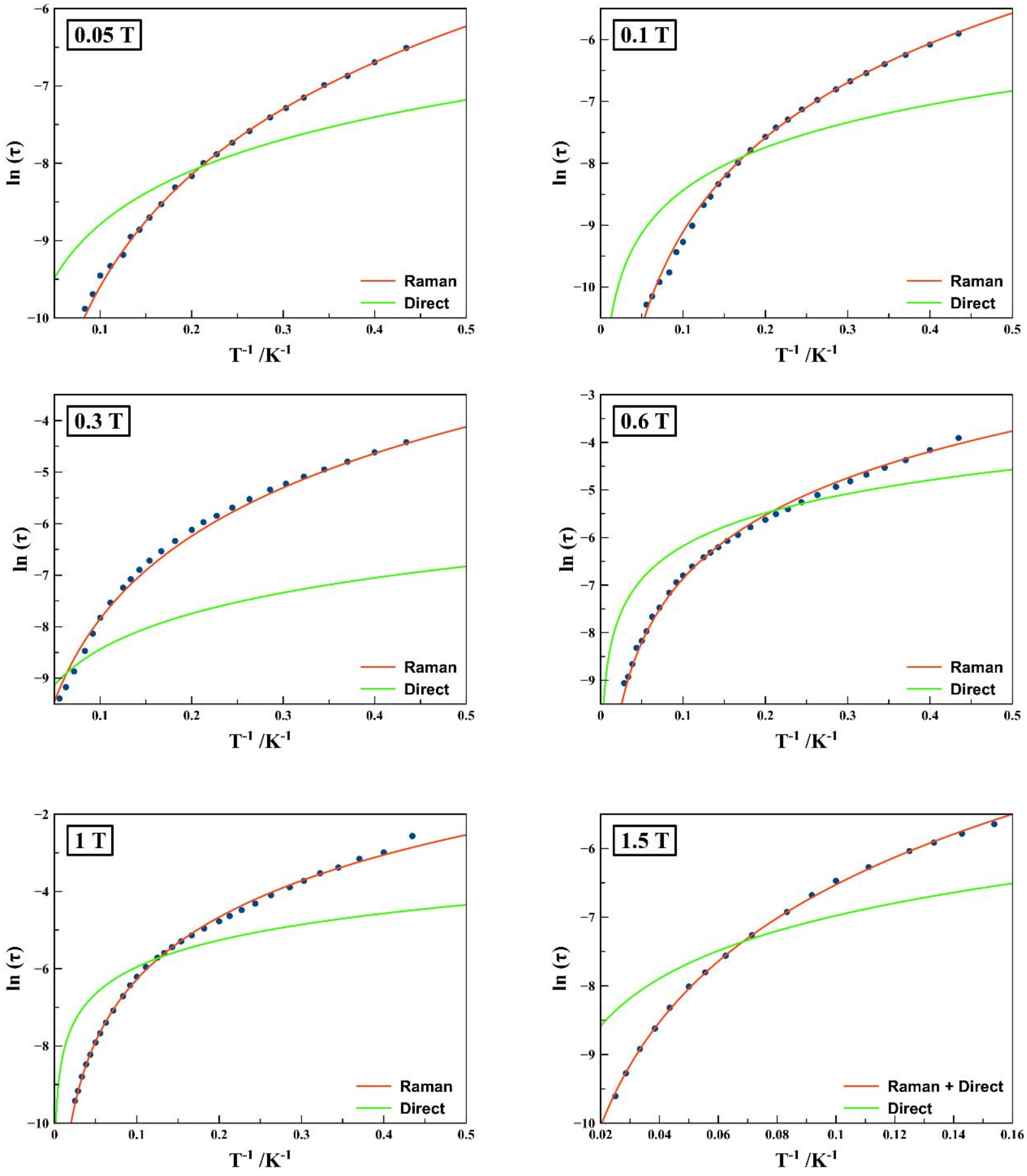
0.05 T
0.1 T
0.3 T
0.6 T
1 T
1.5 T
Raman
Direct
Raman + Direct
ln (τ)
T⁻¹ /K⁻¹

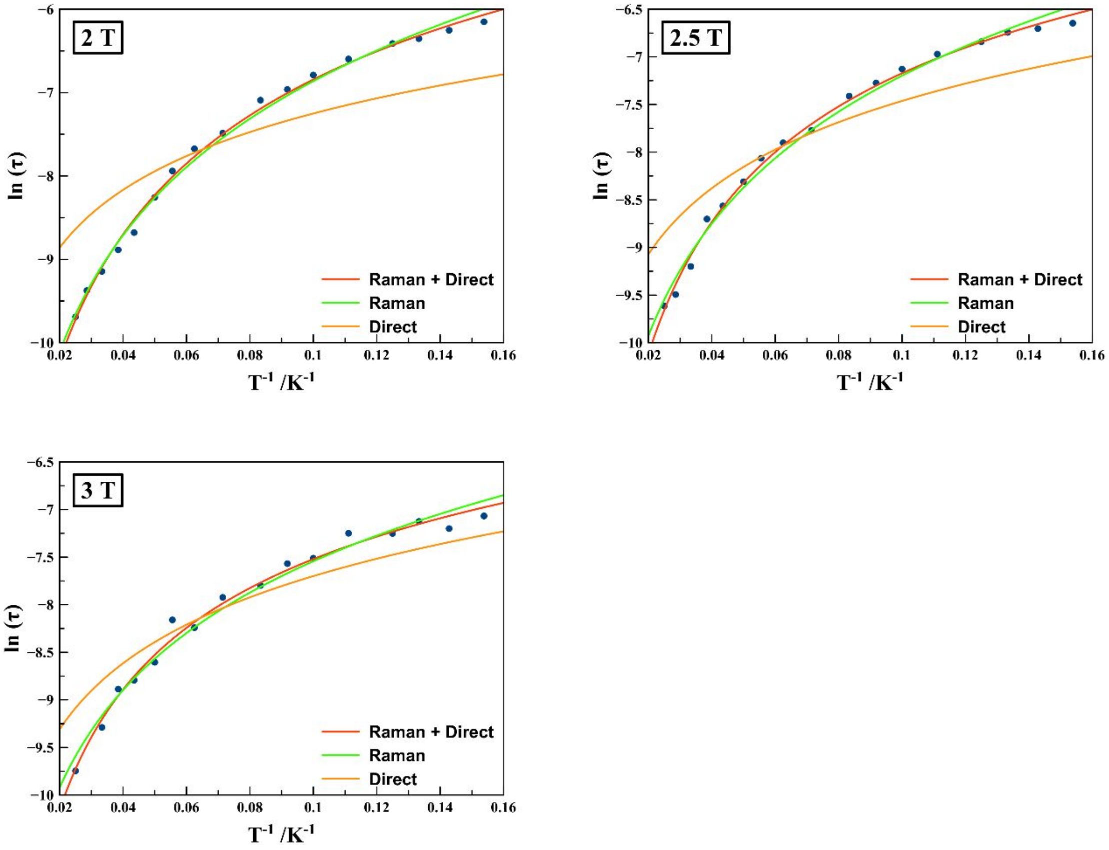

Figure S15. Fitted plots of the temperature dependence of the relaxation times for compound **1** as a function of the applied magnetic field. Solid lines show the best fit of the plots.

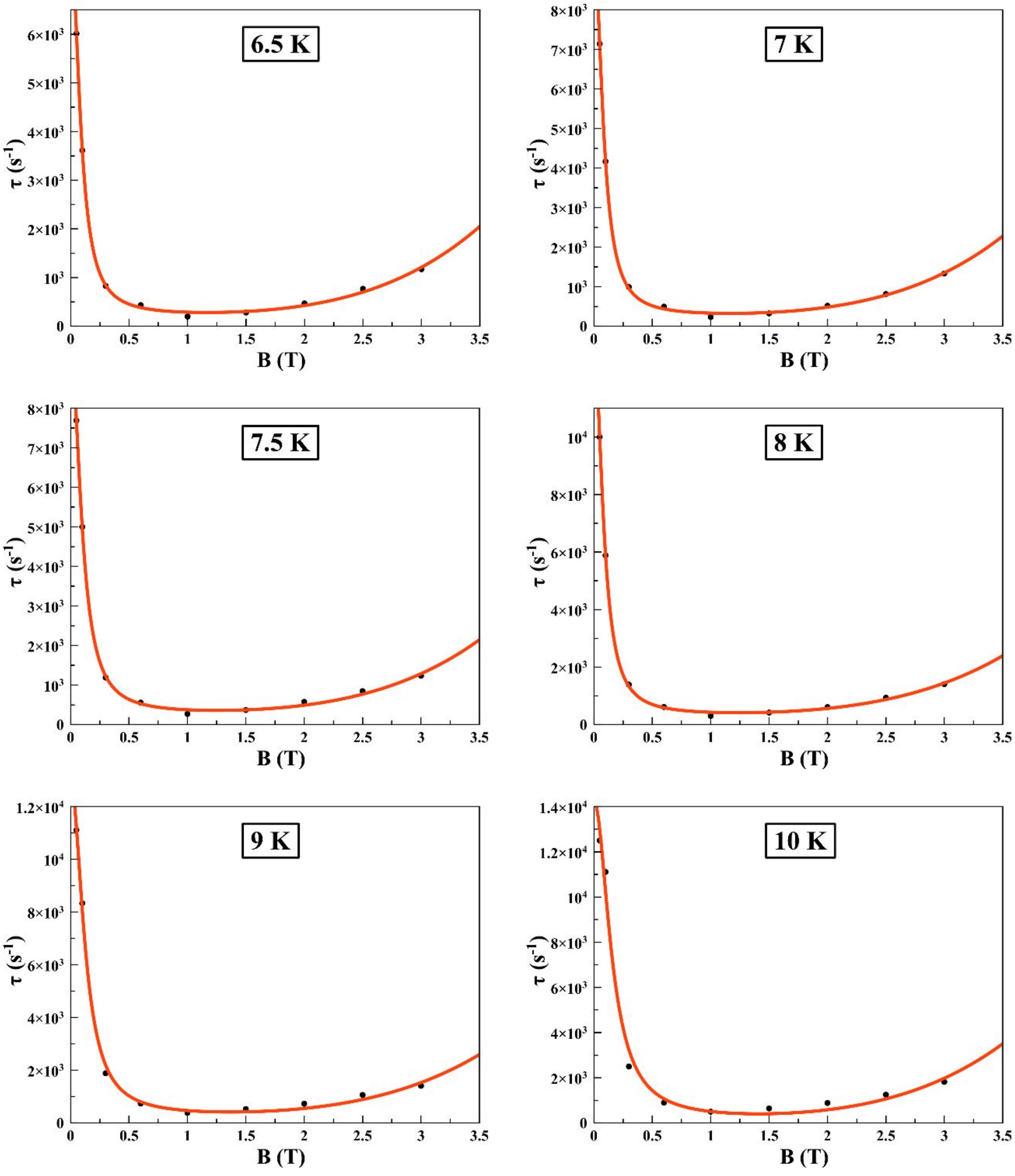
6.5 K
7 K
7.5 K
8 K
9 K
10 K
τ (s^-1)
B (T)

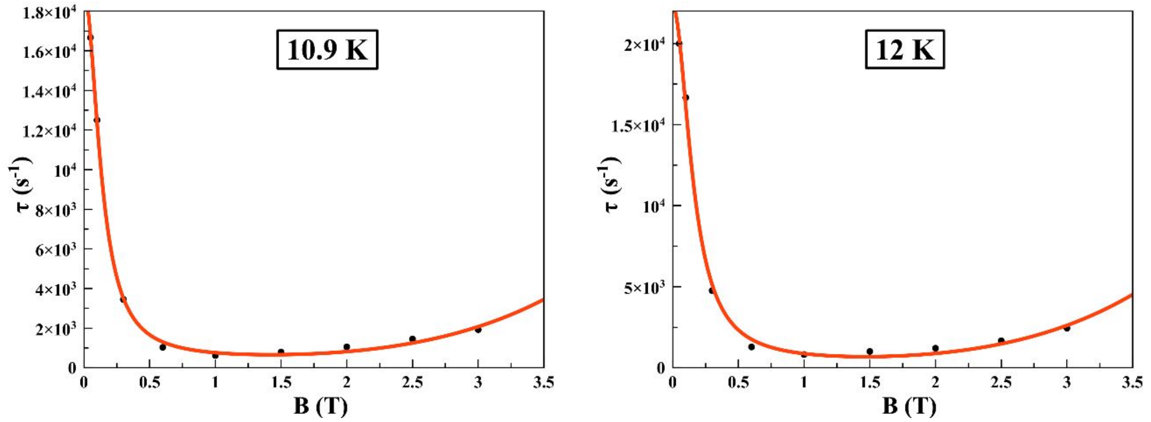

Figure S16. Magnetic field dependence of τ^{-1} extracted from ac susceptibility measurements for compound **1** at different temperatures. Red lines are the best-fits models.

Table S6. Best-fit parameters of the model used to reproduce the field dependence of the magnetization relaxation for **1** (**Equation 2** in the main text).

T (K)	c ($T^{-4}s^{-1}$)	d (ms^{-1})	e (T^{-2})	f (T^{-2})
**6.5**	12.2(9)	7.8(2)	3.4(6)	124(8)
**7**	13.5(8)	9.4(2)	3.5(5)	133(7)
**7.5**	12(1)	9.5(2)	2.6(5)	95(6)
**8**	14(1)	13.1(2)	3.1(4)	129(6)
**9**	16(3)	12.9(4)	1.2(7)	61(8)
**10**	22(9)	14.3(8)	0.4 (1.04)*	39(11)
**10.9**	20(3)	19.9(4)	1.2(4)	55(5)
**12**	27(7)	22.3(7)	0.53(0.53)*	39(5)

*Non-reliable values

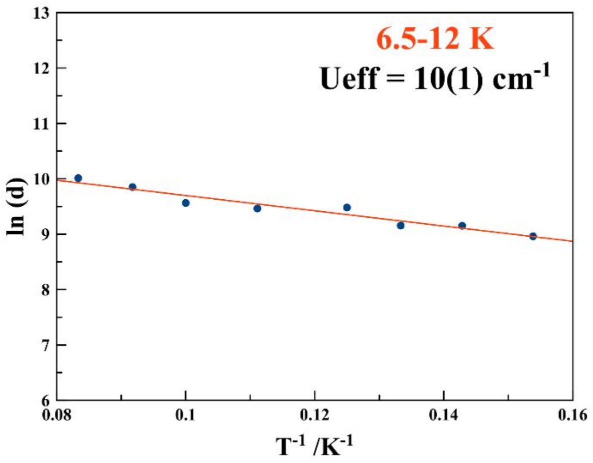

Figure S17. Arrhenius plot of d parameters extracted from the fit of the Brons - van Vleck model.

4- EPR.

4.1 Description of ELDOR-detected NMR (EDNMR)

The pulse sequence for ELDOR-detected NMR is shown in Figure S18a. A high turning angle (HTA) microwave (mw) pulse, with a variable frequency ν_{HTA} burns a hole into an inhomogeneously broadened EPR line and excites electron paramagnetic resonance (EPR) forbidden transitions (green arrows in Figure S18b). The second and third mw pulses are the detection pulses, which give rise to the Hahn echo at a fixed frequency ν_{det}. Nuclear magnetic transitions are excited when the first pulse is resonant with an EPR forbidden transition (at ν_{HTA}) while detecting an EPR allowed transition (red arrows in Figure S18b) (at ν_{det}). Thus, EDNMR spectra are plots of the EPR signal amplitude versus the shift between the two mw frequencies ($\nu_{HTA} - \nu_{det}$).

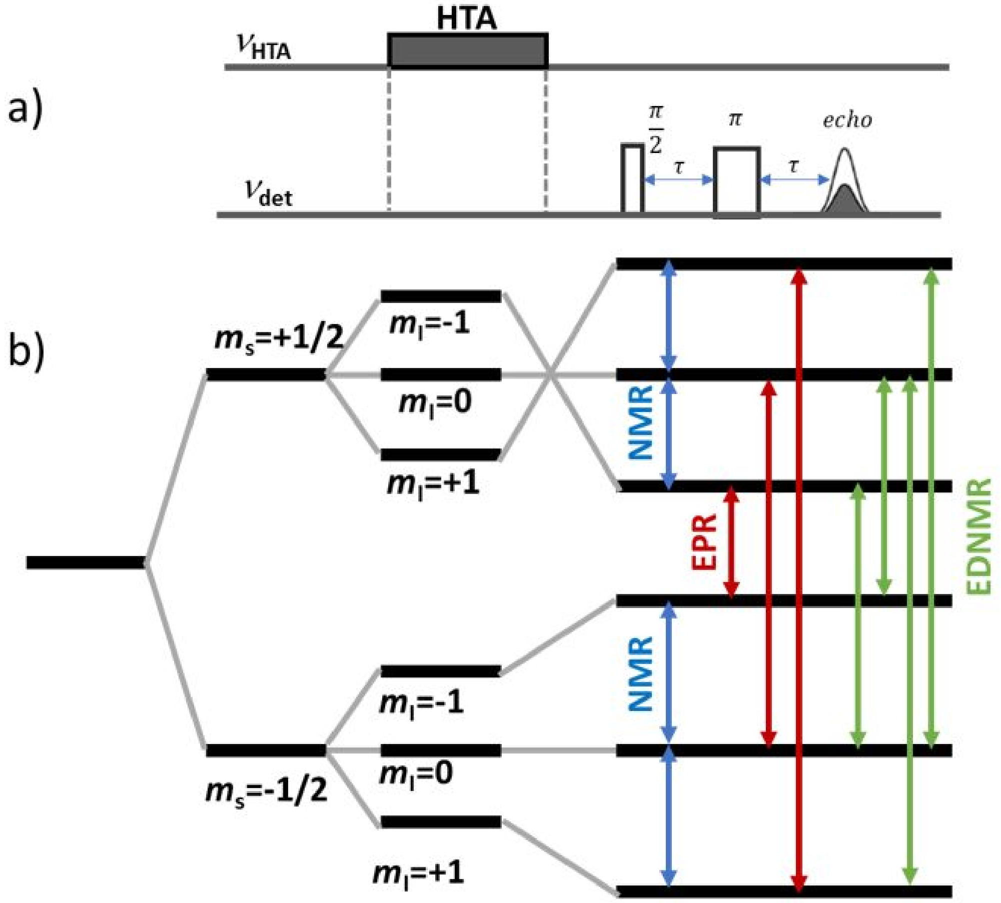

Figure S18 a) Schematic illustration of the EDNMR pulse sequence. b) Energy levels diagram of an *S* = 1/2 and *I* = 1 electron−nuclear system. Blue arrows correspond to the allowed NMR transition, the red arrows to the three allowed EPR transitions with $\Delta m_S = \pm 1$ and $\Delta m_I = 0$. Green arrows correspond to the EDNMR transitions with $\Delta m_S = \pm 1$ and $\Delta m_I = \pm 1$.

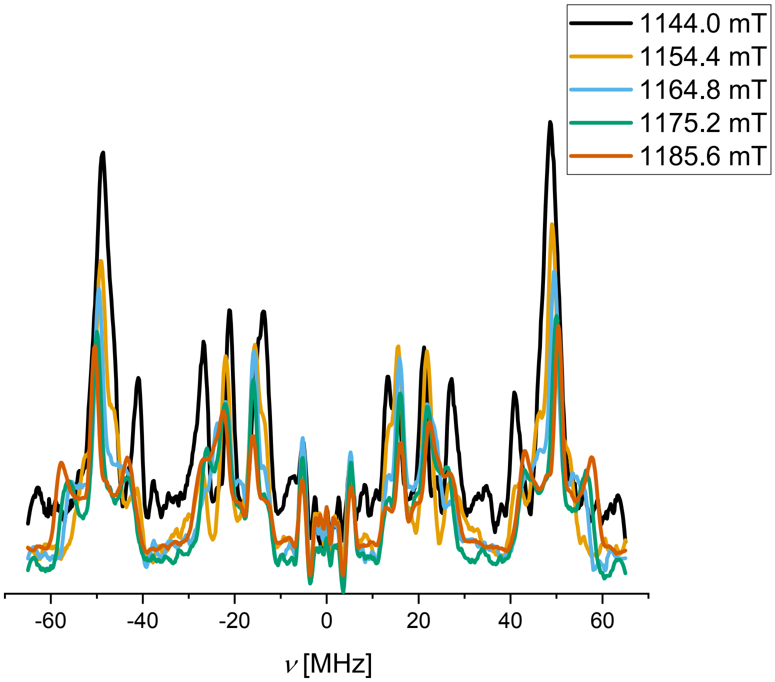

Figure S19. Experimental EDNMR spectra at Q-band frequency and *T* = 20. The central line at zero frequency has been subtracted from the experimental spectra.

Table S7. Spin Hamiltonian parameters for silver (II) nitrogen complexes. Hyperfine coupling values are given in units of MHz

				Ag			N			Ref
	g_x	g_y	g_z	A_x	A_y	A_z	A_x	A_y	A_z	
Ag(II)TTP	2.037	2.037	2.108	88	88	174	63	61	79	2
Ag(II) porphyrin	2.035	2.057	2.076	168	161	273	64	66	71	3
1	2.025	2.025	2.11	110	110	168	33	33	42	This work
							35	36	47	

Table S8. Spin-Hamiltonian parameters used for the simulation of the EDNMR spectra.

Nucleus	A_x [MHz]	A_y [MHz]	A_z [MHz]	*Q* [MHz]	η	(α,β,γ) [°]	(α,β,γ) [°]
$^{14}N(1)$	33	33	42	3	0	(0, 90, 0)	(0, 90, 0)
$^{14}N(2)$	35	36	47	3	0.0	(0, 90, 0)	(0, 90, 0)
^{1}H	3	6	19	-	-	(0, 90, 0)	-

5- ^{14}N Hyperfine coupling and determination of spin density over the coordinating nitrogen ligands.

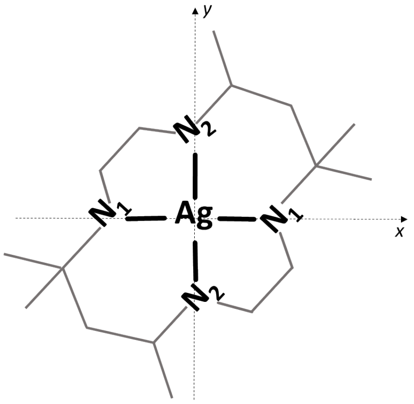

Scheme S1 Reference system with the Ag^{2+} ion placed at the origin

From the observation of the hyperfine structure and determination of the isotropic (a_{iso}) and dipolar (T) hyperfine couplings, the electronic spin distribution in the molecular fragment can be obtained. To do so it is important that the *hf* interaction with several nuclei in the molecule are observed. Then, with the knowledge of a_{iso} and T for the atomic species, and assuming that the *hf* interaction at a given nucleus is proportional to the electron spin density at that nucleus, one can obtain the spin population in s-type orbitals ρ_s and p-type orbitals ρ_p [4].

For a σ orbital involving an arbitrary ligand atom N in the first coordination sphere and pointing along the metal−ligand direction (Scheme S1), the principal values of the ligand hyperfine tensor are given by:

$$A_x = a_{iso}(2s) + A_{||}(2p_x) + A_x(D) + A_{\perp}(2p_z)$$

$$A_y = a_{iso}(2s) + A_{\perp}(2p_x) + A_y(D) + A_{\perp}(2p_z)$$

$$A_z = a_{iso}(2s) + A_{\perp}(2p_x) + A_z(D) + A_{||}(2p_z)$$

In the above expressions, the first terms are the isotropic hyperfine coupling (s character of the σ bond), the second terms give the through-bond anisotropic hyperfine coupling (p character of the σ bond), and the third terms describe the through-space dipolar interaction. The last terms are introduced to consider the slightly orthorhombic hyperfine tensor found experimentally, suggesting a contribution of out-of-plane π bonding.

The experimental tensor can thus be decomposed as follows

A = a_{iso}**I + T + D + T'** (Equation S1**)**

where a_{iso} is the isotropic component (Fermi contact) of the hyperfine interaction **I** the unitary matrix ***D*** the dipolar through space tensor and ***T*** and ***T'*** the through bond dipolar tensors**.**

Considering the crystallographic Ag-N distances (*R*=2.156(2)-2.160(1) Å), the dipolar through space interaction can be neglected in first approximation. Assuming the coordinate reference system shown in Scheme S1 the experimental tensor can therefore be decomposed as follows:

$$A = \begin{vmatrix} A_x & & \\ & A_y & \\ & & A_z \end{vmatrix} = a_{iso} + \begin{vmatrix} 2T & & \\ & -T & \\ & & -T \end{vmatrix} + \begin{vmatrix} -T & & \\ & -T & \\ & & 2T \end{vmatrix}$$

Which for the experimentally derived data gives:

$$A(N_1) = \begin{vmatrix} 42 & & \\ & 33 & \\ & & 33 \end{vmatrix} = 36 + \begin{vmatrix} 6 & & \\ & -3 & \\ & & -3 \end{vmatrix} + \begin{vmatrix} 0 & & \\ & 0 & \\ & & 0 \end{vmatrix}$$

$$A(N_2) = \begin{vmatrix} 35 & & \\ & 47 & \\ & & 36 \end{vmatrix} = 39.3 + \begin{vmatrix} -4 & & \\ & +8 & \\ & & -4 \end{vmatrix} + \begin{vmatrix} -0.3 & & \\ & -0.3 & \\ & & +0.6 \end{vmatrix}$$

For an unpaired electron (free electron, g_e=2.0023) on a ^{14}N-nucleus with a unitary spin population (ρ_s =1) in an s-type orbital, one would observe an isotropic hyperfine coupling constant of a_0=1540 MHz. [5] If the electron resides in a p-type orbital one would observe a uniaxial hyperfine constant of $b_0 = -127.22 \times \frac{2}{5} = -50.9$ MHz. [5] Including a correction for the difference in the *g* values, the spin populations in *s*-type and *p*-type orbitals can thus be estimated as:

$$\rho_s = \frac{A_{iso}}{a_0}\frac{g_e}{g_{iso}};\ \rho_p = \frac{T}{b_0}\frac{g_e}{g_{iso}}$$

$$\rho_s(\mathrm{N}_1) = \frac{36}{1540}\ \frac{2.0023}{2.053} = 0.023$$

$$\rho_p(\mathrm{N_1}) = \frac{-3}{-50.9}\frac{2.0023}{2.053} = 0.060$$

$$\rho_s(\mathrm{N_2}) = \frac{39.3}{1540}\;\frac{2.0023}{2.053} = 0.025$$

$$\rho_p(\mathrm{N_2}) = \frac{-4}{-50.9}\frac{2.0023}{2.053} + \frac{-0.3}{-50.9}\frac{2.0023}{2.053} = 0.084$$

Corresponding to a total spin density repartition over the coordinating N nuclei of approximately 38%.

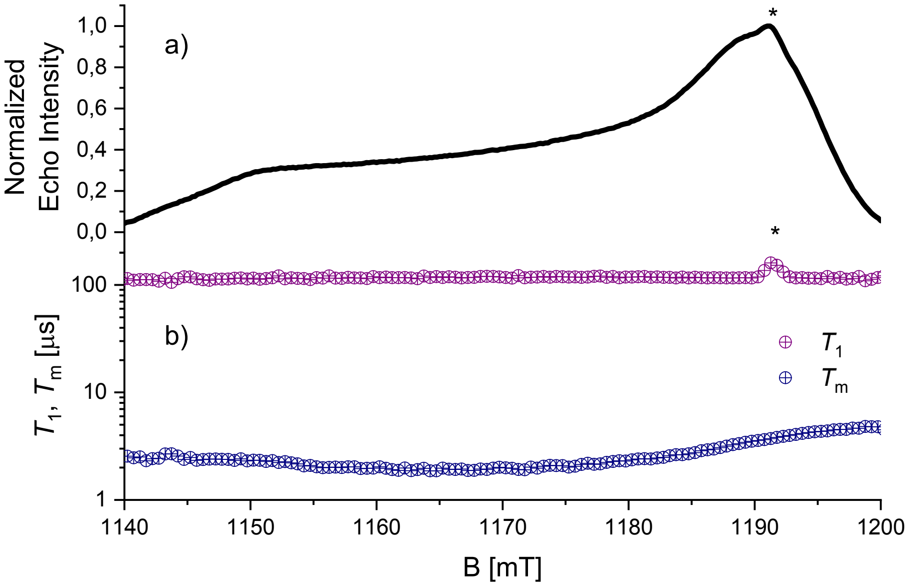

Figure S20. (a) 33.8 GHz ESE detected EPR spectrum of 0.5 mM [Ag^{II}(m-CTH)(BF_4)$_2$] in 1:3 toluene:dichloromethane at 40 K. (b) Resonant field dependence of T_1 and *T*m at 40K. The asterisk indicates a spurious radical signal with long T_1 relaxation.

Table S9. Best fit parameters obtained using **Equation 1** of main text to reproduce the *T* dependence of T_1 for compound **1**.

A_{dir}	8.71×10^{-6} MHz
A_{loc}	1.93×10^{-6} MHz
ν_{loc}	39.16 cm^{-1}

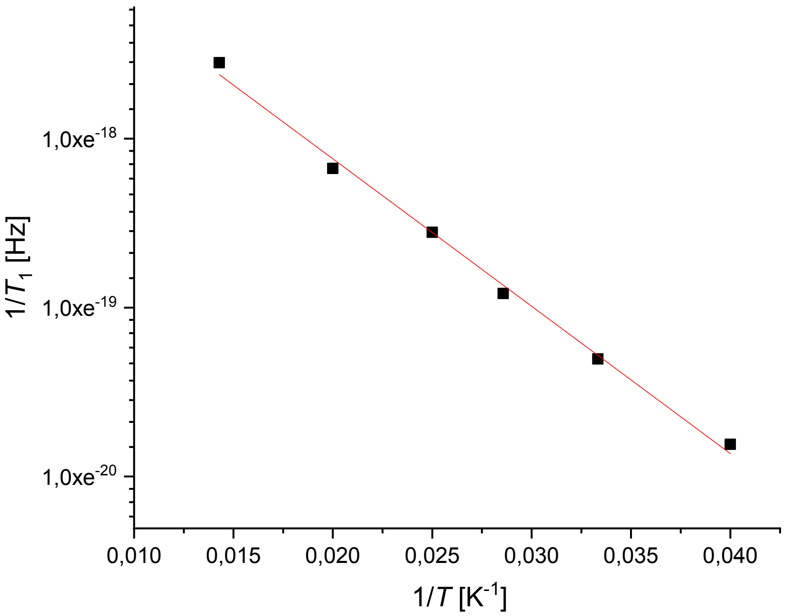

Figure S21. Linear plot of $1/T_1$ (ln scale) against $1/T$. Red line linear fit. The high temperature (25-70 K) T_1 values have been analysed following the procedure adopted by Eaton and Eaton for Ag(II)TTP porphyrin assuming an Orbach process. Rather than an Orbach process (not consistent with an S=1/2 system), we interpret this analysis akin an Arrhenius plot.[6] The linear fit yields a frequency of 60 cm^{-1} and a coupling factor of 16 Hz. The energy barrier for Ag(II)CTH is slightly smaller than Ag(II)TTP, this is consistent with the more flexible nature of the CTH ligand. However, a quantitative comparison between the two systems is hampered by the different operational frequencies (Q-band vs X-band) at which the data were recorded.

REFERENCES

[1] SHAPE v.2.0. Lunell, M.; Casanova, D.; Cirera, J.; Alemany, P.; Alvarez, S. Barcelona **2010**. The program can be obtained by request to the authors.

[2] Brown, T. G.; Hoffman, B. M. 14N, 1H, and metal ENDOR of single crystal Ag (II)(TPP) and Cu (II)(TPP). *Molecular Physics*, **1980**, *39(5)*, 1073-1109.

[3] Singh, A. K.; Usman, M.; Sciortino, G.; Garribba, E.; Rath, S. P. Through-Space Spin Coupling in a Silver (II) Porphyrin Dimer upon Stepwise Oxidations: AgII··· AgII, AgII··· AgIII, and AgIII··· AgIII Metallophilic Interactions. *Chemistry–A European Journal*, **2019**, *25(43)*, 10098-10110.

[4] J. A. Weil; J. R. Bolton; Wertz, J. E. *Electron Paramagnetic Resonance – Elementary Theory and Practical Applications*, Wiley, New York, **1994**.

[5] Fitzpatrick, J. A. J.; Manby, F. R.; Western, C. M. The interpretation of molecular magnetic hyperfine interactions. J. *Chem. Phys.,* **2005**, 122, 084312.

[6] Jackson C. E. et al. A reaction-coordinate perspective of magnetic relaxation Chem. Soc. Rev., **2021**,50, 6684-6699
